# Loss of Myonuclei and Transcriptional Activity During Diaphragm Atrophy in Critically Ill Patients

**DOI:** 10.1002/jcsm.70228

**Published:** 2026-02-15

**Authors:** Wout J. Claassen, Marloes van den Berg, Zhong Hua Shi, Rianne. J. Baelde, Sylvia Bogaards, Luuk Bonis, Heleen Hakkeling, Arezou Bamyani, Gerben J. Schaaf, Albertus Beishuizen, Chris Dickhoff, Reinier A. Boon, Leo Heunks, Tyler J. Kirby, Coen A. C. Ottenheijm

**Affiliations:** ^1^ Department of Physiology Amsterdam UMC, location VUmc Amsterdam the Netherlands; ^2^ Department of Cellular and Molecular Medicine University of Arizona Tucson Arizona USA; ^3^ Center for Lysosomal and Metabolic Diseases Erasmus University Medical Center Rotterdam the Netherlands; ^4^ Intensive care Center Medisch Spectrum Twente Enschede the Netherlands; ^5^ Department of Surgery Amsterdam UMC, location VUmc Amsterdam the Netherlands; ^6^ Department of Intensive Care Medicine Radboudumc Nijmegen the Netherlands; ^7^ Division of Cardiovascular Medicine, Department of Internal Medicine University of Kentucky Lexington Kentucky USA

**Keywords:** critical care, diaphragm weakness, mechanical ventilation

## Abstract

**Background:**

Diaphragm weakness frequently develops in critically ill patients and is explained by a combination of atrophy and myofiber dysfunction. Myofibers are large syncytial cells maintained by a population of myonuclei, which provide gene transcripts to a finite fiber volume, termed the myonuclear domain. Myonuclear number is a determinant of transcriptional capacity and therefore critical for diaphragm and peripheral muscle regeneration after critical illness. Changes in myonuclear number in myofibers undergoing atrophy have not been investigated in mechanically ventilated ICU patients, but they are of potential clinical importance. Our objective was to investigate if and how myonuclear number changes in the diaphragm of mechanically ventilated ICU patients and whether changes are associated with myofiber atrophy and clinical parameters.

**Methods:**

We used a combination of transcriptomics, immunohistochemistry and confocal microscopy to study myonuclear alterations in the diaphragm and quadriceps biopsies from mechanically ventilated ICU patients (*n* = 24) and non‐critically ill patients (*n* = 10).

**Results:**

Compared to control patients, myonuclear number and myonuclear domain were reduced in critically ill patients with diaphragm myofiber atrophy (*n* = 14) (myonuclear number per mm of 133 [92–183] vs. 92 [83–105], *p* = 0.03 (slow myofibers) and 149 [118–189] vs. 88 [69–109], *p* = 0.004 (fast myofibers); myonuclear domain size was 44 [34–51] vs. 29 pL, *p* = 0.004 (slow myofibers) and 41 [39–48] vs. 27 pL, *p* = 0.001 (fast myofibers) of control patients and ICU patients with atrophy, respectively). Increased intrinsic apoptotic pathway activation was identified as a mechanism underlying myonuclear removal (percentage of apoptotic myonuclei of 0.64 [0.60–0.84] and 0.95 [0.84–1.2], *p* = 0.015 and increased percentage of activated caspase‐3 positive myonuclei of 2,5 [1.6–3.3] vs. 5.7 [4.3–11], *p* = 0.001 in control patients and ICU patients with atrophy, respectively). Total transcriptional activity in myofibers decreased with myonuclear loss (RNA‐Pol‐2 Ser5 fluorescence intensity per fibre of 2.6 [2.2–3.3] vs. 5.8 [3.1–6.7] AU, *p* = 0.036 in control patients and ICU patients with atrophy, respectively). Furthermore, muscle stem cell number was reduced in the patients with diaphragm atrophy (PAX7 positive nuclei per myofiber of 0.10 [0.09–0.11] vs. 0.05 [0.04–0.07], *p* = 0.002 in control patients and ICU patients with atrophy, respectively). No correlation was found between myonuclear loss and duration or mode of mechanical ventilation.

**Conclusions:**

We identified myonuclear loss due to intrinsic apoptotic pathway activation as a potential mechanism underlying diaphragm atrophy in mechanically ventilated patients. The loss of myonuclei may contribute to impaired regeneration of myofibers after critical illness. Duration and mode of mechanical ventilation are not the major drivers of these modifications.

## 1 Introduction

Weakness of the diaphragm frequently develops in ventilated critically ill patients. This contributes to difficult weaning, which is associated with increased morbidity, mortality and healthcare costs [1]. Furthermore, it can lead to physical disability and long‐term impairment in intensive care survivors [2]. Diaphragm weakness is caused by a combination of atrophy and dysfunction of the remaining contractile material, leading to weakness of muscle cells (i.e., myofibers). In recent years, several mechanisms underlying contractile dysfunction have been identified, including changes in myosin conformation [3, 4]. The mechanisms underlying atrophy are not well understood.

Myofibers are large syncytial cells that are maintained by a population of post‐mitotic myonuclei. Each myonucleus provides gene transcripts to a finite fibre volume, termed the myonuclear domain [5]. During changes in muscle mass, myofibers' nuclear number may change [6]. In several pathological states resulting in myofiber atrophy, apoptosis regulates myonuclear number [7]. In the diaphragm of ventilated rodents and brain‐dead organ donors, myonuclear loss and upregulated apoptosis were identified to underlie diaphragm atrophy [8, 9], but these pathways have not yet been investigated in ventilated critically ill patients. Brain‐dead organ donors and rodents do not represent the complexity of critically ill patients, with respect to ventilation strategies, inflammatory profile and metabolic stress [8]. For example, in contrast to brain‐dead organ donors and most mechanically ventilated rodent models, the diaphragm is not fully unloaded in mechanically ventilated ICU patients due to the use of support‐mode mechanical ventilation. It is critical to investigate whether apoptosis and myonuclear loss occur in mechanically ventilated ICU patients as, during weaning and further recovery, the diaphragm needs to regain mass and function for which nuclear number and their transcriptional activity are important [10]. Finally, if myonuclear apoptosis plays a role in critical illness‐associated diaphragm weakness, inhibiting the underlying mechanism may be a promising therapeutic avenue, potentially improving clinical outcome [10].

Thus, our objective was to study apoptotic pathways in the diaphragm of mechanically ventilated critically ill patients. Furthermore, we studied myonuclear number and myonuclear domain size in myofibers isolated from diaphragm biopsies. It was evaluated whether changes in myonuclear number and myonuclear domain were related to duration and mode of mechanical ventilation. In the first part of the study, we compared the biopsies of ICU patients with those of controls. To study whether these changes are dependent on atrophy, in the second part, we divided the ICU patients into two groups: one with and one without atrophy. Additionally, we obtained quadriceps biopsies from another cohort of ventilated critically ill patients to investigate whether the mechanism we describe also affects peripheral muscles. Some of the results of these studies have been previously reported in conference abstracts and as a preprint (Supporting Information S1: S1, S2).

## 2 Methods

For further details on the applied methods, see the online supplement.

### 2.1 Patients, Diaphragm Biopsies

Diaphragm muscle biopsies were obtained from invasively ventilated critically ill patients (*ICU* patients, *n* = 24) and patients undergoing elective lung surgery for early‐stage lung malignancy (*Control* patients, *n = 10*). Exclusion criteria for both groups were chronic obstructive pulmonary disease (≥ GOLD stage III), congestive heart failure, neuromuscular diseases, chronic metabolic disorders, pulmonary hypertension, chronic use of corticosteroids (> 7.5 mg/day for at least 3 months) and more than 10% weight loss within the last 6 months. The exclusion criteria were similar for all experimental groups. Patients' characteristics are presented in Table 1. Diaphragm biopsies were collected from 24 critically ill patients who underwent laparotomy or thoracotomy for a clinical indication. The biopsies were collected from the left anterolateral part of the zone of apposition of the diaphragm. The biopsy protocol was approved by the institutional review board at Amsterdam UMC (location VUmc), the Netherlands. Written informed consent was obtained from the patients or their legal representative. To study whether myonuclear changes were dependent on atrophy, the ICU group was divided into two groups for sub‐analysis: one with atrophy (*N* = 14) and one without atrophy (*N* = 10).

**TABLE 1:** Clinical characteristics of patients that underwent diaphragm biopsies.

Characteristic	Control (*n* = 10)	ICU patients (*n* = 24)	ICU A+ (*n* = 14)	ICU A− (*n* = 10)	*p*
Age (years)	67 [60–72]	66 [50–73]	65 [55–71]	60 [49–69]	0.698
Male (%)	7 (70)	15 (63)	7 (50)	8 (80)	
BMI (Kg/m^2^)	28 [24–32]	25 [21–28]	25 [21–28]	26 [21–28]	0.281
APACHE‐3	—	73 [47–111]	78 [53–149]	75 [67–89]	0.762
Ventilation (hours)	1.5 [0.9–2.0]	66 [44–193]	63 [45–121]*	90 [18–223]*	< 0.001
Controlled ventilation (hours)	2.0 [1–2.3]	64 [32–127]*	64 [34–132]*	55 [16–140]*	< 0.001
Myofiber CSA (μm^2^)	2469 [1708–2973]	1736 [1250–3035]	1341 [1007–1667]*	3159 [2798–4294]*	< 0.001
Medical history, *n* (%)					
Smoking	3 (30)	13 (54)	10 (71)	3 (30)	0.072
COPD ≤ G2	3 (30)	3 (13)	2 (14)	1 (10)	0.506
Other lung disease	2 (20)	1 (4)	1 (7)	0 (0)	0.259
Cardiac	1 (10)	3 (13)	2 (14)	1 (10)	0.996
Arterial vascular disease	2 (20)	14 (58)*	12 (86)*	2 (20)	0.010
Hypertension	3 (30)	9 (38)	6 (43)	3 (30)	0.860
CKD	1 (10)	2 (8)	2 (14)	0 (0)	0.535
T2DM	0 (0)	2 (8)	0 (0)	2 (20)	0.179
Hypothyroidism	1 (10)	2 (8)	2 (14)	0 (0)	0.716
Malignancy lung	7 (70)	1 (4)*	0 (0)*	1 (10)*	< 0.001
Malignancy other	2 (20)	2 (1)	2 (1)	0 (0)	0.466
Medication					
Steroids	—	18 (75)	11 (79)	9 (90)	0.715
Neuromuscular blockers	—	12 (50)	8 (57)	4 (40)	0.433
Vasopressors	—	19 (79)	10 (71)	9 (90)	0.495

*Note:* Data displayed as Median [IQR]. *p*‐values calculated with Kruskal–Wallis or chi‐squared tests BMI: Body Mass Index. *P*‐values of continuous data calculated with one‐way analysis of variance or Kruskal–Wallis tests, depending on distribution of the data. *p*‐values of categorical data calculated with chi‐squared test.

Abbreviations: A‐a gradient = alveolar–arterial gradient, APACHE = Acute Physiology and Chronic Health Evaluation, BMI: Body Mass Index; CID = Chronic Inflammatory Disease, CKD = Chronic Kidney Disease, COPD = Chronic Obstructive Pulmonary Disease, CSA = Cross‐Sectional Area, MVhr: duration of mechanical ventilation before biopsy, P/F‐ratio = arterial partial pressure of oxygen (PaO_2_) divided by the inspired oxygen concentration (FiO_2_), T2DM = Type 2 Diabetes Mellitus.

* indicates a significant difference with the control group calculated with post hoc tests.

### 2.2 Patients, Quadriceps Biopsies

Quadriceps biopsies of critically ill patients (*N* = 10) were obtained in the context of a separate study that has been filed in the Clinical Trial Register under #NCT03231540 and was approved by the Medical Ethical Committee of VU Medical Center, Amsterdam, the Netherlands. Informed consent was obtained from the patient or a legal representative.

### 2.3 Single Myofiber Microscopy

Single myofibers were manually isolated from the biopsies. Myonuclear number, myofiber volume, myonuclear morphology and RNA‐polymerase‐II Ser5 fluorescence were determined using immunofluorescence labeling in combination with confocal microscopy.

### 2.4 RNA‐Sequencing and Immunohistochemistry

RNA was extracted using a commercially available kit and sequenced using a NextSeq500 (Illumina). Serial cryosections for immunohistochemistry were cut from the biopsies and stained to study myonuclear number, myonuclear domain, markers for apoptosis and satellite cell content.

### 2.5 Statistical Analysis

Normality of the distribution of the studied variables was assessed visually on normal probability plots. Log transformation was performed if necessary. To compare the difference between ICU patients and control patients, student's *t*‐test or Mann–Whitney U was applied for non‐repeat measurements; and linear mixed model with patients as the random factor was applied for measurements involving technical replicates in all human samples. For linear mixed models, Greenhouse–Geisser correction was applied to adjust for potential lack of sphericity. For the comparison of three groups or more, one‐way analysis of variance (ANOVA) or Kruskal–Wallis tests were performed with Tukey's or Dunn's post hoc tests, depending on the distribution of the data. We used a two‐sided significance level of 5% for all analyses. Unless otherwise noted, data are expressed as mean (± standard error), median [interquartile range] or frequencies (percentage), as appropriate.

## 3 Results

Clinical characteristics of ventilated patients (*N* = 24) are shown in Table 1. The biopsies were compared to the biopsies of 10 control patients. The ICU group was later divided into two sub‐groups for further analysis: one with atrophy *(N* = 14) and one without atrophy *(N =* 10). The groups were matched for age, body mass index and sex (Table 1). Due to the limited size of the biopsies, not every biopsy was used in every experiment. Table S1 details which biopsies were used for each experiment.

### 3.1 Transcriptomics Reveals Upregulation of Apoptotic Pathways in the Diaphragm

**FIGURE 1:**
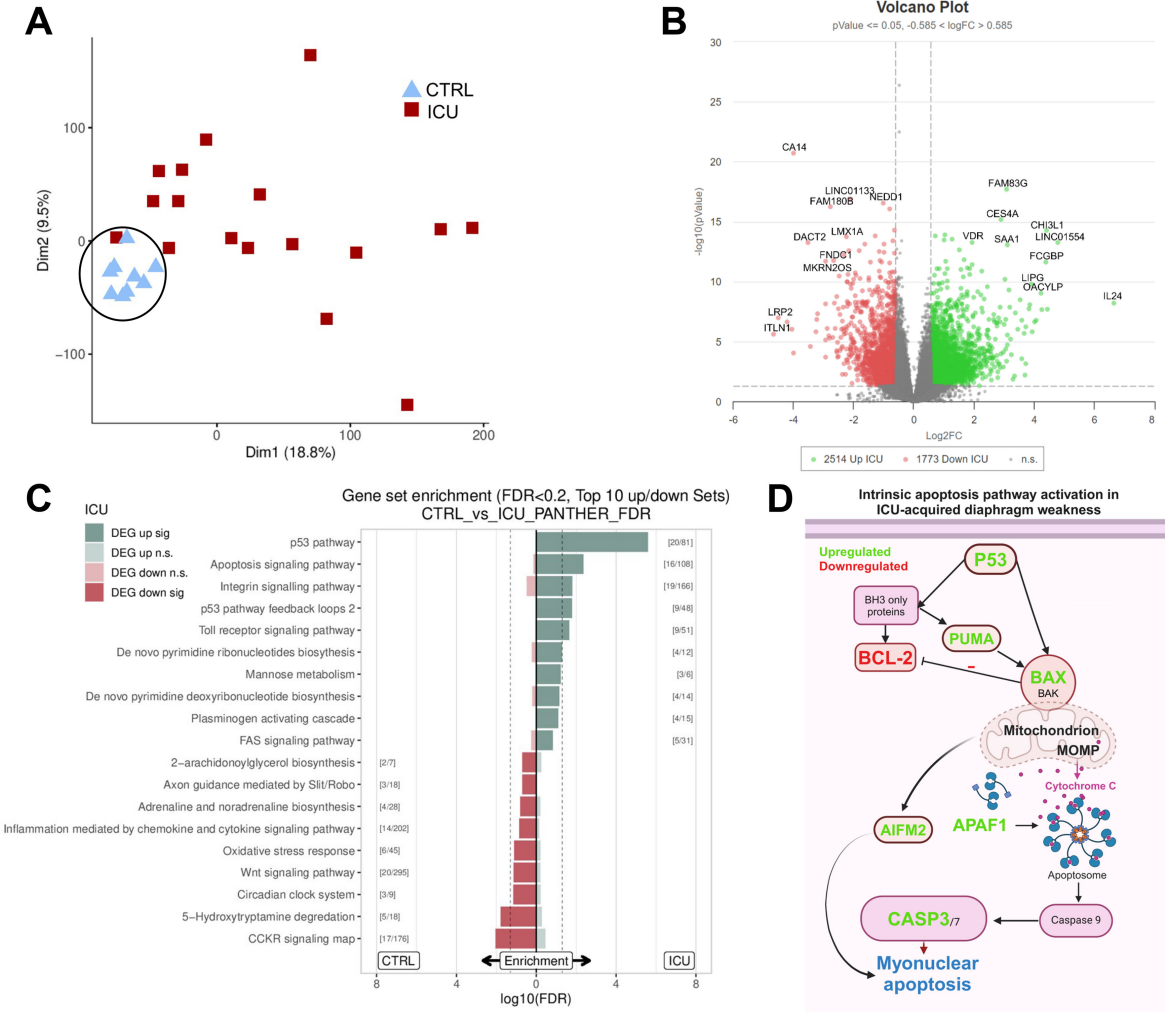
RNA sequencing of the diaphragm of mechanically ventilated ICU patients. (A) Principal component analysis of sequencing results. Note the clustering of the samples within the CTRL group while this clustering is absent in the ICU group. This may be due to heterogeneity of patient characteristics within the ICU group. CTRL *N* = 8; ICU *N* = 17. (B) Volcano plot and (C) PANTHER gene set enrichment analysis. Top10 gene sets or pathways enriched for up‐ or down‐regulated genes of one database (dashed line: *p*‐value = 0.05). Only shows pathways that are not significant for both directions (up/down) at the same time to identify on/off situations. Significant gene set enrichment is defined by the false discovery rate. CTRL *N* = 8; ICU *N* = 17. (D) Schematic of the differentially expressed genes that are involved in the intrinsic apoptotic pathway. Genes in green are significantly upregulated and genes in red are significantly downregulated in ICU patients.

To investigate the mechanisms underlying diaphragm weakness and atrophy, we performed next‐generation RNA‐sequencing of whole tissue samples cut from the biopsies (*N =* 9 controls, *N* = 17 ICU patients). Table S2 shows the clinical characteristics of the patients included in this experiment. Principal component analysis (PCA) summarizes RNA‐sequencing data into the most important patterns or components. Clear clustering was present in the control patients, while the ICU group had more variable clustering (Figure 1A), which means that gene expression patterns in the control patients were more similar than those in the ICU patients. We identified 2977 differentially expressed genes (1741 upregulated in the ICU group and 1236 downregulated) (Figure 1B) between control and ICU patients. A heatmap of the top 50 differentially expressed genes was generated (Figure S1A). Next, we performed Panther pathway enrichment analysis; the dashed lines closest to the middle represent the significance threshold for differentially expressed pathways (Figure 1C). Consistent with our hypothesis of increased apoptosis in ICU patients, the top upregulated pathways in the ICU group were the p53, apoptosis and integrin signalling pathways. In Figure S1B, the differentially expressed genes that are involved in the apoptotic pathway are shown. Note the significantly up or downregulated pathways that reach past the significance threshold (dotted vertical line). The extrinsic apoptotic pathway gene set was not significantly up or downregulated (Figure 1C). We did not find differential expression of the extrinsic apoptotic pathway associated death receptor ligands FASL (CD95L) or TRAIL (Supporting Information S1: S3). Furthermore, we did not find differential expression of FADD, an important component of the death‐inducing signalling complex, which assembles in response to extrinsic apoptotic stimuli and activates caspases associated with the extrinsic apoptotic pathway (Supporting Information S1: S4). Thus, in mechanically ventilated ICU patients, the p53 and intrinsic apoptosis pathways are upregulated (Figure 1D) providing a potential mechanism underlying weakness and atrophy.

### 3.2 Caspase‐3‐Mediated Apoptosis of Myonuclei

**FIGURE 2:**
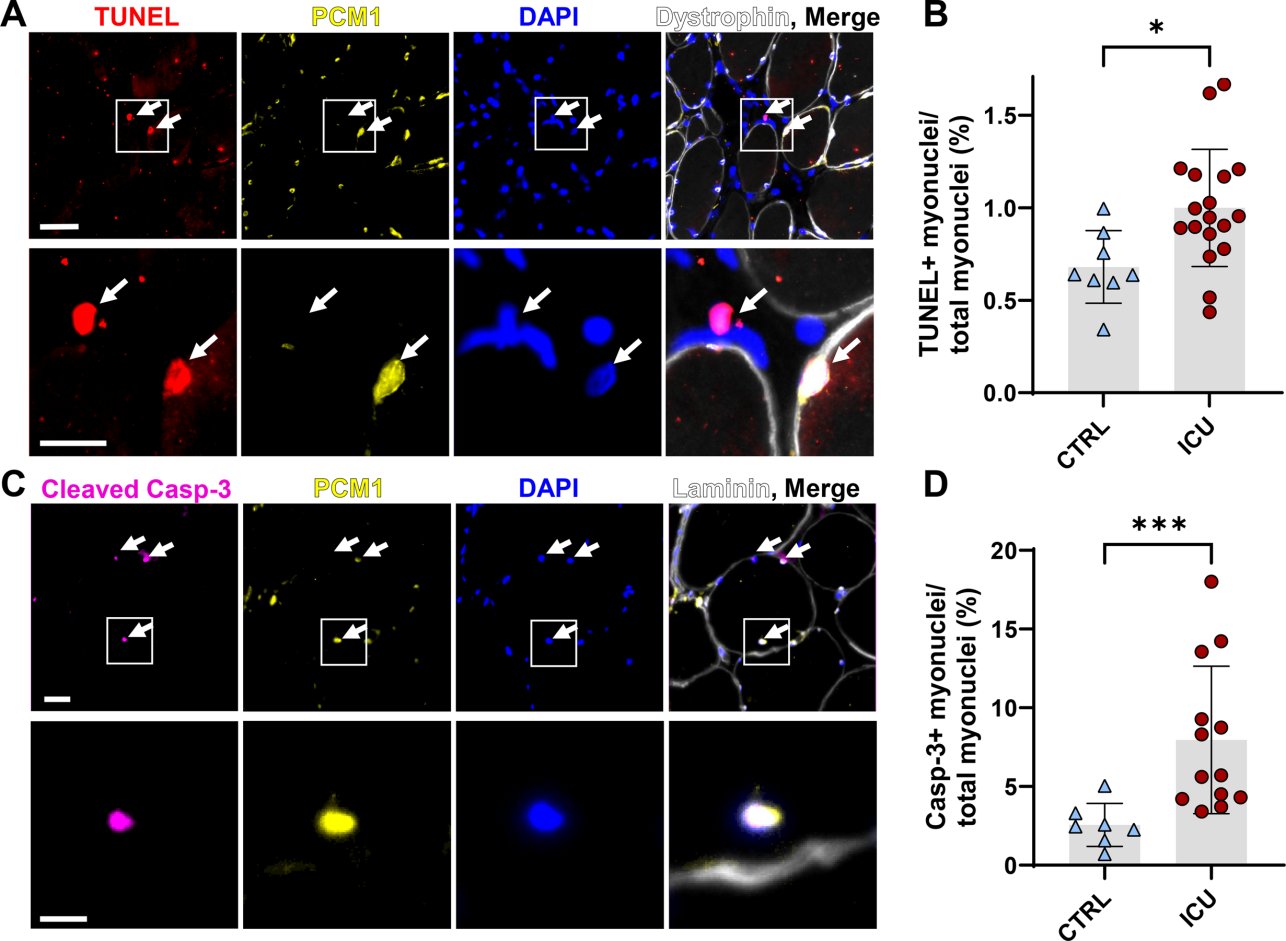
Caspase‐3 mediated apoptosis as a mechanism underlying myonuclear loss in the diaphragm of ICU patients. (A) Representative images of diaphragm muscle cross‐sections stained with TUNEL assay, PCM1 antibody, Dystrophin antibody and DAPI. Nuclei with a TUNEL‐positive signal were designated as apoptotic myonuclei when they were PCM1 positive and were located within the dystrophin barrier. Note the difference in PCM1 immunoreactivity of the TUNEL+ non‐myonucleus (left white arrow) and myonucleus (right white arrow). Scale bar top row is 50 μm, scale bar bottom row is 20 μm. (B) Quantification of TUNEL index, calculated as the percentage of TUNEL‐positive myonuclei. Total myonuclear count was determined by counting PCM1‐positive nuclei. The grey bar represents the mean ± standard deviation within the groups of patients. Each coloured symbol represents the value of a single patient. CTRL *N* = 8; ICU *N* = 18. Significance level was calculated using an unpaired *t*‐test. (C) Representative images of diaphragm muscle cross‐sections stained with Cleaved caspase‐3 antibody, PCM1 antibody, Laminin antibody and DAPI. Nuclei with a Cleaved Caspase‐3 positive signal were designated as apoptotic myonuclei when they were PCM1 positive and were located within the laminin barrier. Scale bar top row = 50 μm, scale bar bottom row = 20 μm. (D) Quantification of activated caspase‐3 index, calculated as the percentage of activated caspase‐3‐positive myonuclei. Total myonuclear count was determined by counting PCM1‐positive nuclei. The grey bar represents the mean ± standard deviation within the groups of patients. Each coloured symbol represents the value of a single patient. CTRL *N* = 7; ICU *N* = 14. Significance level was calculated using a Mann–Whitney U test. ICU A+ = CU group with atrophy, ICU A− = ICU group without atrophy, CTRL = Control group, * = *p* < 0.05, ** = *p* < 0.01.

As bulk‐tissue RNA‐sequencing revealed marked upregulation of the apoptotic pathway, we next performed analyses on muscle cross‐sections to allow for the identification of different cell types that may be undergoing apoptosis. We used pericentreolar‐material‐1 (PCM1) [11] and localization within the immunolabelled dystrophin/laminin barrier as markers for myonuclei, as previously it was suggested that much of the apoptosis that occurs during muscle atrophy can be attributed to the non‐myonuclear cell pool [11]. Tables S3 and S4 show the characteristics of the patients included in these experiments. The percentage of myonuclei undergoing apoptosis was determined using terminal deoxynucleotidyl transferase dUTP nick end labeling (TUNEL) of double‐stranded DNA breaks (*N* = 8 controls, *N* = 18 ICU patients) (Figure 2A). Representative images of TUNEL labeling of a DNase‐I treated diaphragm section (positive control) are shown in Figure S2. The mean TUNEL‐index (number of TUNEL‐positive myonuclei divided by the total number of myonuclei) in the ICU group was almost double that of the control group (Figure 2B). There was no difference in TUNEL‐index for other cell types (Figure S3A). Furthermore, we used an antibody for cleaved (i.e., activated) caspase‐3 to investigate the role of caspase‐3 mediated nuclear apoptosis (*N* = 7 Controls, *N* = 14 ICU patients) (Figure 2C) [12]. The mean activated caspase‐3 index (number of cleaved caspase‐3‐positive myonuclei divided by the total number of myonuclei) was ~3‐fold higher in the ICU group (Figure 2D). Caspase‐3‐index for other cell types was also higher in the ICU group (Figure S3B).

Next, to investigate whether increased apoptosis may play a role in peripheral muscles of critically ill patients, we obtained quadriceps muscle biopsies of a different cohort of mechanically ventilated critically ill patients with comparable age, BMI and APACHE‐score and compared them to healthy controls (*N* = 5 controls, *N* = 10 ICU patients). Tables 2, S5 and S6 show the clinical characteristics of these groups. Note that the duration of mechanical ventilation was shorter in the quadriceps cohort (median of 39 vs. 66 h; quadriceps vs. diaphragm group). In the control groups, there was no difference in the activated caspase‐3 index between the diaphragm and quadriceps (Figure S4E). However, in the ICU‐Quadriceps group, the activated caspase‐3 index of both myonuclei and other cell types was almost double that of the CTRL‐Quadriceps group (Figure S4B,C), but still more than two‐fold lower than in the diaphragm (Figure S4D). These findings should be interpreted with caution, as other factors, such as differences in disease aetiology due to neuromuscular blockade or use of steroids, may have influenced the findings in the quadriceps. The mean myofiber CSA was similar in the ICU‐Quadriceps and CTRL‐Quadriceps groups (Figure S4F), indicating that in the quadriceps of mechanically ventilated ICU patients, caspase‐3 activation may occur before the onset of atrophy.

**TABLE 2:** Characteristics of individual patients of quadriceps studies.

Patient	Age (y)	Sex	BMI (kg/m^2^)	ICU diagnosis	APACHE‐4 score	Duration of MV (h)
1	57	M	26	Trauma	139	33
2	81	M	25	Trauma	94	44
5	30	F	27	Trauma	76	51
6	32	M	22	Trauma	76	51
8	84	M	23	Sepsis	130	28
9	60	M	25	Trauma	94	17
10	24	M	24	Trauma	63	79
11	71	M	29	Trauma	40	14
14	71	M	28	Resp. failure	97	49
15	66	M	20	Resp. failure	104	14
C01	21	M	23	—	—	—
C02	21	M	22	—	—	—
C03	57	M	26	—	—	—
C04	51	F	23	—	—	—
C05	58	M	32	—	—	—

Thus, both our transcriptomic and imaging data support a mechanism where caspase‐3‐mediated apoptosis is increased during critical illness‐associated diaphragm weakness.

### 3.3 Decreased Myonuclear Number and Myonuclear Domain in Atrophic Diaphragm Fibres

To investigate whether the upregulation of apoptosis in diaphragm myonuclei resulted in a reduced myonuclear number, we counted myonuclei stained with a lamin A/C antibody in 8–12 myofibers per biopsy that were randomly selected and manually isolated. This method allows for a precise determination of myonuclear number per myofiber volume. Myonuclear domain was determined by dividing myofiber volume by myonuclear number. We performed immunofluorescent staining of myonuclear marker PCM1 on the same single myofiber preparations of a subset of ICU and control patients to test whether the presence of nuclei other than myonuclei could have influenced myonuclear number. This experiment revealed that the proportion of PCM1‐positive myonuclei within the isolated myofibers (*N* = 3 controls, *N* = 3 ICU patients) (Table S7) was > 98% in both groups, showing that the number of non‐myonuclei included in the preparations did not impact myonuclear number determined by lamin A/C staining (Figure S5). Fast and slow‐twitch fibres were identified by immunoreactivity for fast‐twitch myosin heavy chain isoform and data were segregated according to fibre type.

**FIGURE 3:**
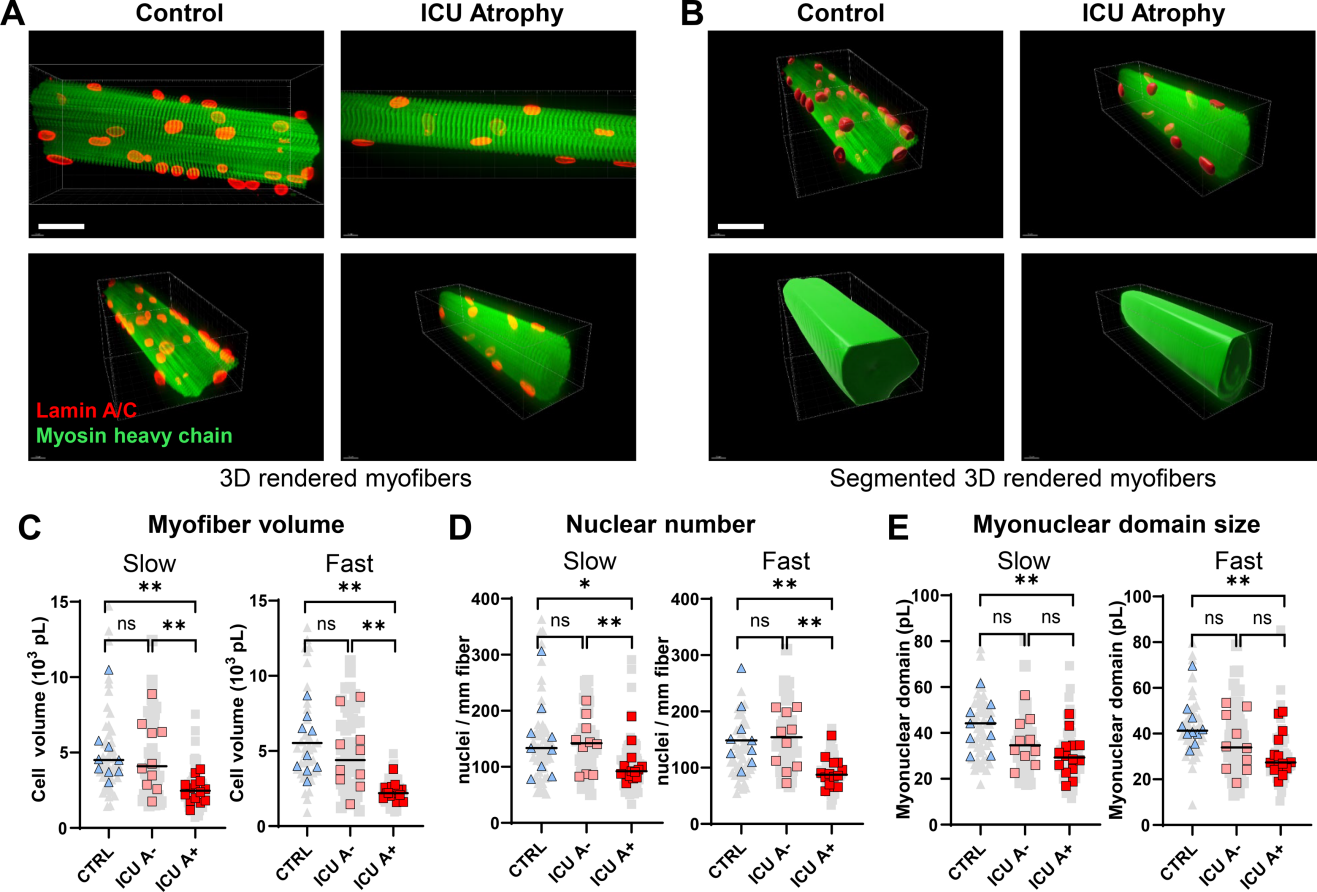
Reduced number of myonuclei in atrophic diaphragm fibres of critically ill patients. (A) Representative images of single muscle fibres from control (left) and ICU (right) patients. Myofibers are immunofluorescently labelled for lamin A/C (red) and myosin heavy chain (green) Scale bar = 60 μm (B) Representative images of 3D rendered single muscle fibres, with segmentation in 3D. Scale bar = 60 μm. (C) Quantification of myofiber volume, calculated as volume per mm fibre was normalized to a sarcomere length of 2.5 μm. Every grey dot represents the value of a single muscle fibre, and the coloured symbols represent the mean values of a single patient. Slow‐twitch fibres: ICU A+ *N =* 14, *n* = 84; *N =* 10, *n =* 50; CTRL *N =* 10, *n =* 48. Fast‐twitch fibres: ICU A+ *N =* 14, *n =* 56; ICU A− *N =* 10, *n =* 48; CTRL *N =* 10, *n =* 46. (D) Quantification of myonuclear number. Every grey dot represents the value of a single muscle fibre, and the coloured symbols represent the mean values of a single patient. Black bars indicate the median of the whole group. Slow‐twitch fibres: ICU A+ *N =* 14, *n =* 84; ICU A− *N =* 10, *n =* 50; CTRL *N =* 10, *n =* 48. Fast‐twitch fibres: ICU A+ *N =* 14, *n =* 56; ICU A− *N =* 10, *n =* 48; CTRL *N =* 10, *n =* 46. (E) Quantification of myonuclear domain size. Every grey dot represents the value of a single muscle fibre, and the coloured symbols represent the mean values of a single patient. Significance levels were calculated using linear mixed models with the patients as the random factor. Black bars indicate the median of the whole group. Slow‐twitch fibres: ICU A *N =* 14, *n =* 84; ICU A− *N =* 10, *n =* 50; CTRL *N =* 10, *n =* 48. Fast‐twitch fibres: ICU A+ *N =* 14, *n =* 56; ICU A− N = 10, *n =* 48; CTRL *N =* 10, *n =* 46. ICU A+ = ICU group with atrophy; ICU A− = ICU group without atrophy; CTRL = Control group. *denotes *p* < 0.05; **denotes *p* < 0.01. *N =* number of patients, *n =* number of analysed myofibers.

Myofiber volume and myonuclear domain size were lower in the ICU group, but nuclear number was not significantly lower (Figure S6). We hypothesized that we did not observe a difference in myonuclear number due to the presence of patients in the ICU group with a myofiber volume similar to that of the controls, e.g., without myofiber atrophy. Therefore, to investigate whether myonuclear number was reduced specifically in ICU patients with diaphragm atrophy, we separated the ICU group into two groups: one with atrophy (ICU with atrophy (A+), *N* = 14) and one without atrophy (ICU without atrophy (A−), *N* = 10). Representative images are shown in Figure 3A,B. To this end, we used myofiber CSA data derived from cryosections (mean size of ~450 myofibers). For the ICU group without atrophy, the lower limit of the CSA was set at the median CSA of the control group (CSA of 2500 μm^2^). To ensure minimal overlap between the groups, the upper limit of the ICU group with atrophy was set at a CSA of 2000 μm^2^. Clinical characteristics of all groups are shown in Tables 1 and S8. There were no significant differences in clinical characteristics between the ICU group with atrophy and ICU group without atrophy.

As expected, the volume of the isolated myofibers in the ICU group with atrophy was ~50% smaller compared to the ICU group without atrophy and control group, and this difference was similar for both fibre types (Figure 3C). In the ICU group with atrophy, myonuclear number was reduced by about 35% in both fast and slow myofibers when compared to the ICU group without atrophy and the control group (Figure 3D). To verify whether our findings in single myofibers were representative of a larger sample, we validated these findings by analysing the number of myonuclei per myofiber in diaphragm muscle cross‐sections with an average size of 498 myofibers per section. Myonuclei were identified using PCM1 labeling in combination with localization within the dystrophin barrier (Figure S7A). In muscle cross‐sections, average myonuclear counts per fibre were reduced by about 39% in the diaphragm group with atrophy compared to the diaphragm control group (Figure S7B), which was consistent with the results from the isolated myofibers (Figure 3D). Next, we counted the myonuclei present in the quadriceps cross‐sections and found that there was no difference in the number of myonuclei per myofiber between the control and ICU patients (Figure S7B). In the isolated diaphragm myofibers, myonuclear domain size was smaller in the ICU group with diaphragm atrophy when compared to the control group, but not when compared to the ICU group without atrophy (Figure 3E). Furthermore, there was no significant correlation between the duration of mechanical ventilation or duration of diaphragm inactivity and myonuclear number, caspase‐3 index, TUNEL‐index and Caspase‐3 RNA–levels (Figure S8), indicating that unloading by the ventilator may not be a major driver of apoptosis. To investigate whether reduced myonuclear numbers are tied to clinical outcomes, we correlated myonuclear number to time to first spontaneous breathing trial and time to liberation from the ventilator (Figure S9A,B), but did not find significant correlations.

In conclusion, myonuclear number is reduced in myofibers in the atrophic diaphragm of mechanically ventilated ICU patients, and myonuclear domain is smaller, indicating relatively more atrophy than myonuclear loss. Furthermore, the reduction in myonuclear number was absent in the diaphragm of ICU patients without diaphragm atrophy. Outcomes related to myonuclear apoptosis did not correlate with the duration of mechanical ventilation, making it likely that other patient characteristics drive this mechanism.

### 3.4 Transcriptomic Profiles of ICU Patients With and Without Atrophy

**FIGURE 4:**
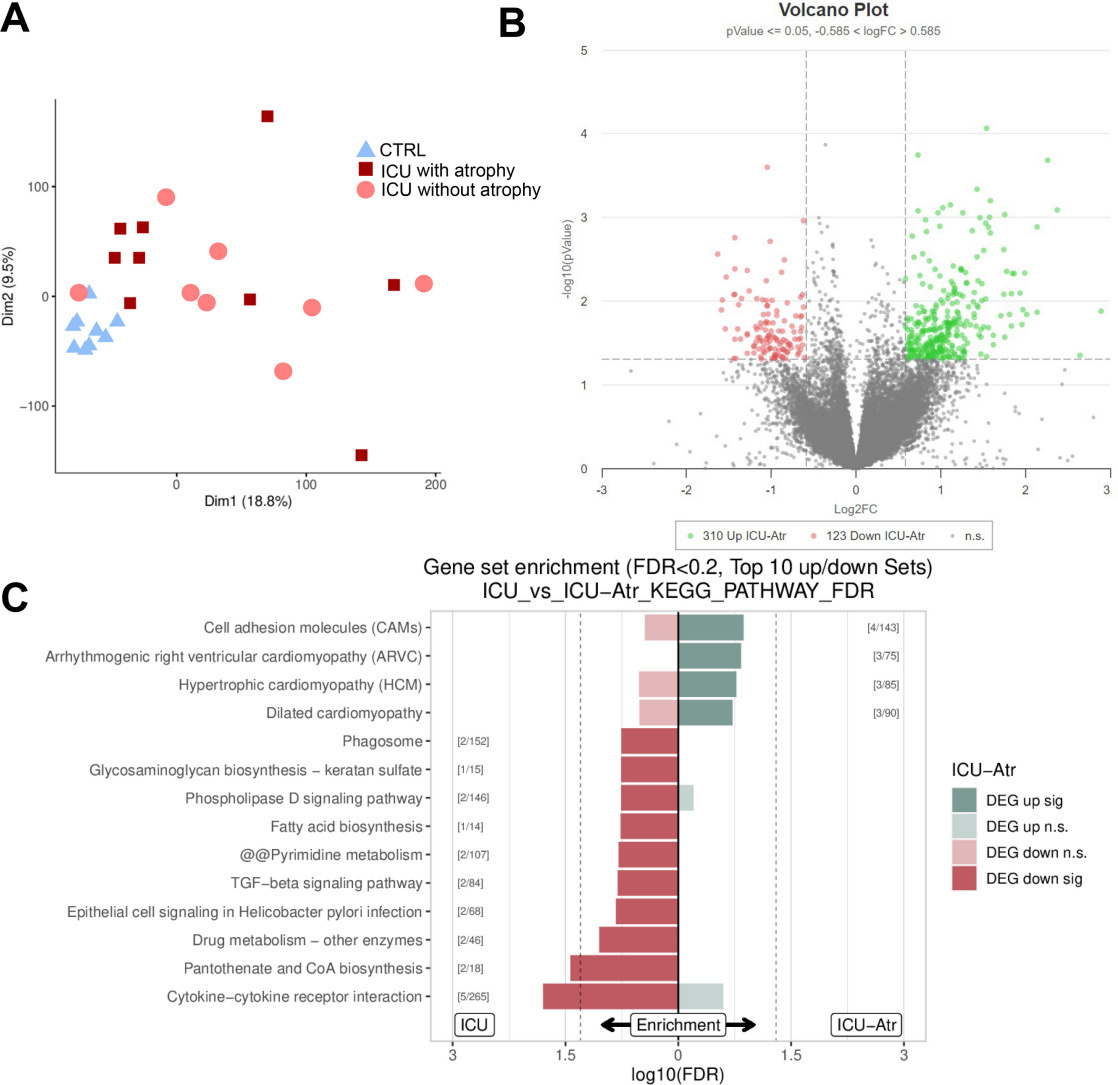
Comparison of transcriptomic profiles of ICU with and without atrophy. (A) Principal component analysis of sequencing results. Note the clustering of the samples within the CTRL group while this clustering is absent in the ICU groups. This may be due to heterogeneity of patient characteristics within both ICU groups. CTRL *N* = 8; ICU‐A+ *N* = 9, ICU *N* = 8. (B) Volcano plot 310 genes were significantly upregulated and 123 genes were significantly downregulated in the ICU‐A+ group. Genes with a significance level *p* < 0.05 and a fold change of > 1.5 were deemed differentially expressed. CTRL *N* = 8; ICU‐A+ *N* = 9, ICU *N* = 8 (C) Gene set enrichment analysis showing the Top10 gene sets or pathways enriched for up‐ or down‐regulated genes of one database (dashed line: *p*‐value = 0.05). Only shows pathways that are not significant for both directions (up/down) at the same time to identify on/off situations. Significant gene set enrichment is defined by the false discovery rate. CTRL *N* = 8; ICU‐A+ *N* = 9, ICU *N* = 8.

Because a reduced myonuclear number was observed in ICA patients with only, we hypothesized that these patients may have a distinct transcriptomic profile compared to ICU patients without atrophy. Analysis of the transcriptomic profiles was adjusted to compare both ICU groups. We did not observe distinct clustering of both ICU groups using principal‐component analysis (Figure 4A). When comparing the ICU group with atrophy to the ICU group without atrophy, 433 genes were differentially expressed (Figure 4B). Comparison of both ICU groups revealed similar expression levels of genes that are associated with the intrinsic apoptotic pathway in both ICU groups (Figure S10), indicating that this pathway is upregulated in patients with and without atrophy.

Finally, pathway analysis revealed upregulation of cytokine‐cytokine receptor interaction in the ICU‐group with atrophy (Figure 4C), indicating that ICU patients with atrophy of the diaphragm may have had more inflammation in the diaphragm. Inflammatory parameters (C‐reactive protein, leucocyte count) in blood and myonuclear number or apoptotic indices did not correlate (Figure S11), suggesting that measurements of systemic inflammation may not reflect the inflammatory status of the diaphragm. In conclusion, ICU patients with diaphragm atrophy likely have upregulated inflammation compared to the patients without atrophy. In the absence of any relationship between apoptosis and duration of mechanical ventilation and diaphragm unloading, localized inflammation or other clinical characteristics may drive atrophy and myonuclear apoptosis.

### 3.5 Diminished Transcriptional Activity of Myofibers in the Atrophic Diaphragm of Mechanically Ventilated ICU Patients

**FIGURE 5:**
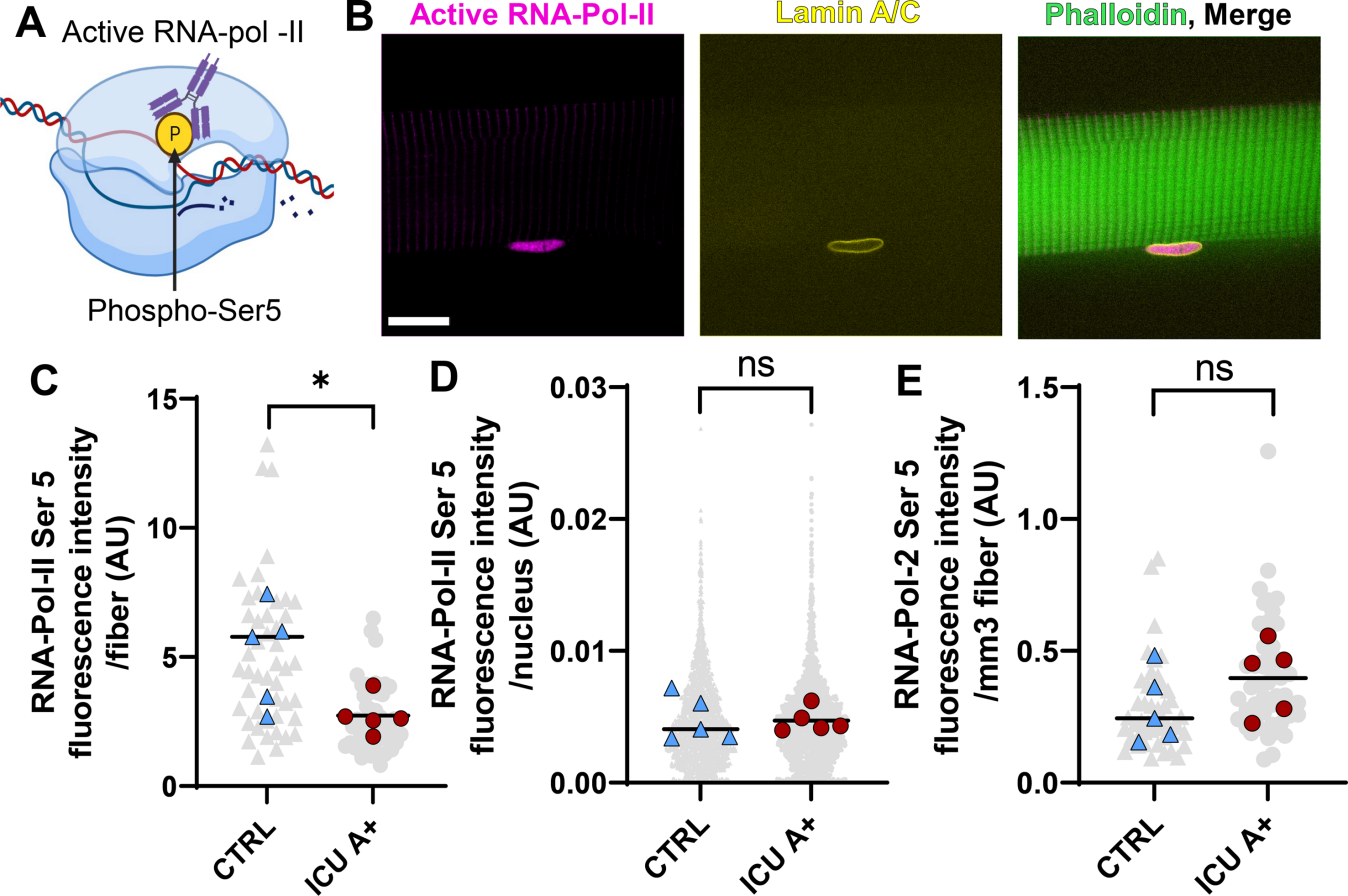
Transcriptional activity of myonuclei in the diaphragm. (A) Schematic of RNA‐polymerase‐II with phosphorylation at Serine 5 with antibody binding to phosphorylated Serine. This phosphorylation occurs when RNA‐Pol‐II starts transcribing DNA into mRNA. (B) Representative images of RNA‐Pol‐II‐Ser5 (magenta), lamin A/C (yellow) and phalloidin (green) labeling of a single Z‐plane of a mounted single muscle fibre. Z‐stacks were used to create 3D renders of fibre segments and total RNA‐Pol‐II Ser5 fluorescence intensity was measured within each myonucleus, using lamin A/C to segment all nuclei in 3D. Scale bar = 20 μm. (C) Total RNA‐Pol‐II‐Ser5 fluorescence intensity was calculated as the sum of the total RNA‐Pol‐II‐Ser5 fluorescence intensity within each nucleus present within three Z‐stacks generated from a single myofiber. CTRL *N = 5* patients, *n* = 48 myofibers; ICU A+ *N =* 5 patients, *n* = 48 myofibers. Each grey symbol represents the value of a single myofiber. Each coloured symbol represents the mean value of a single patient. The black bar represents the mean value within the groups of patients. (D) Total RNA‐Pol‐II‐Ser5 fluorescence intensity per segmented nucleus. CTRL *N =* 5 patients, *n* = 2817 nuclei; ICU A+ *N = 5* patients, *n* = 2235 nuclei. Each grey symbol represents the value of a single nucleus. Each coloured symbol represents the mean value of a single patient. The black bar represents the mean value within the groups of patients. (E) Total RNA‐Pol‐II‐Ser5 fluorescence intensity per myofiber volume was calculated as the sum of the total RNA‐Pol‐II‐Ser5 fluorescence intensity within each nucleus present within the myofiber divided by the myofiber volume. CTRL *N = 5* patients, *n* = 48 myofibers; ICU A+ *N = 5* patients, *n* = 48 myofibers. Each grey symbol represents the value of a single myofiber. Each coloured symbol represents the mean value of a single patient. The black bar represents the mean value within the groups of patients. Significance level was calculated using linear mixed models with the patients as the random factor. ICU A+ = ICU group with atrophy; CTRL = Control group; *denotes *p* < 0.05; **denotes *p* < 0.01.

Myonuclear number has been shown to influence myonuclear transcription, with transcription levels increasing with decreasing nuclear number [13]. Thus, we aimed to determine whether the reduced myonuclear number resulted in changes in myonuclear transcription in ICU patients. Furthermore, transcriptional activity may be downscaled with the reduced myonuclear domain size in ICU patients with diaphragm atrophy because the cellular volume to regulate is smaller. Total transcriptional activity per fibre, calculated by summation of the fluorescence intensity of RNA‐pol‐II Ser5 labeling within each nucleus in the myofiber, was on average almost two‐fold lower in the ICU A+ group compared to the control group (Figure 5C). This was explained by the lower myonuclear number in ICU myofibers because the fluorescence intensity of RNA‐Pol‐II Ser5 per nucleus did not differ between the groups (Figure 5D). After normalization to fibre volume, the difference in transcriptional activity per fibre was absent (Figure 5E). Thus, based on these findings, insufficient transcriptional activity of individual nuclei is unlikely to contribute to atrophy, whereas the reduced myonuclear number in the ICU group with atrophy may reduce total transcriptional activity per myofiber.

### 3.6 Decreased Muscle Stem Cell Number in the Diaphragm of ICU Patients

**FIGURE 6:**
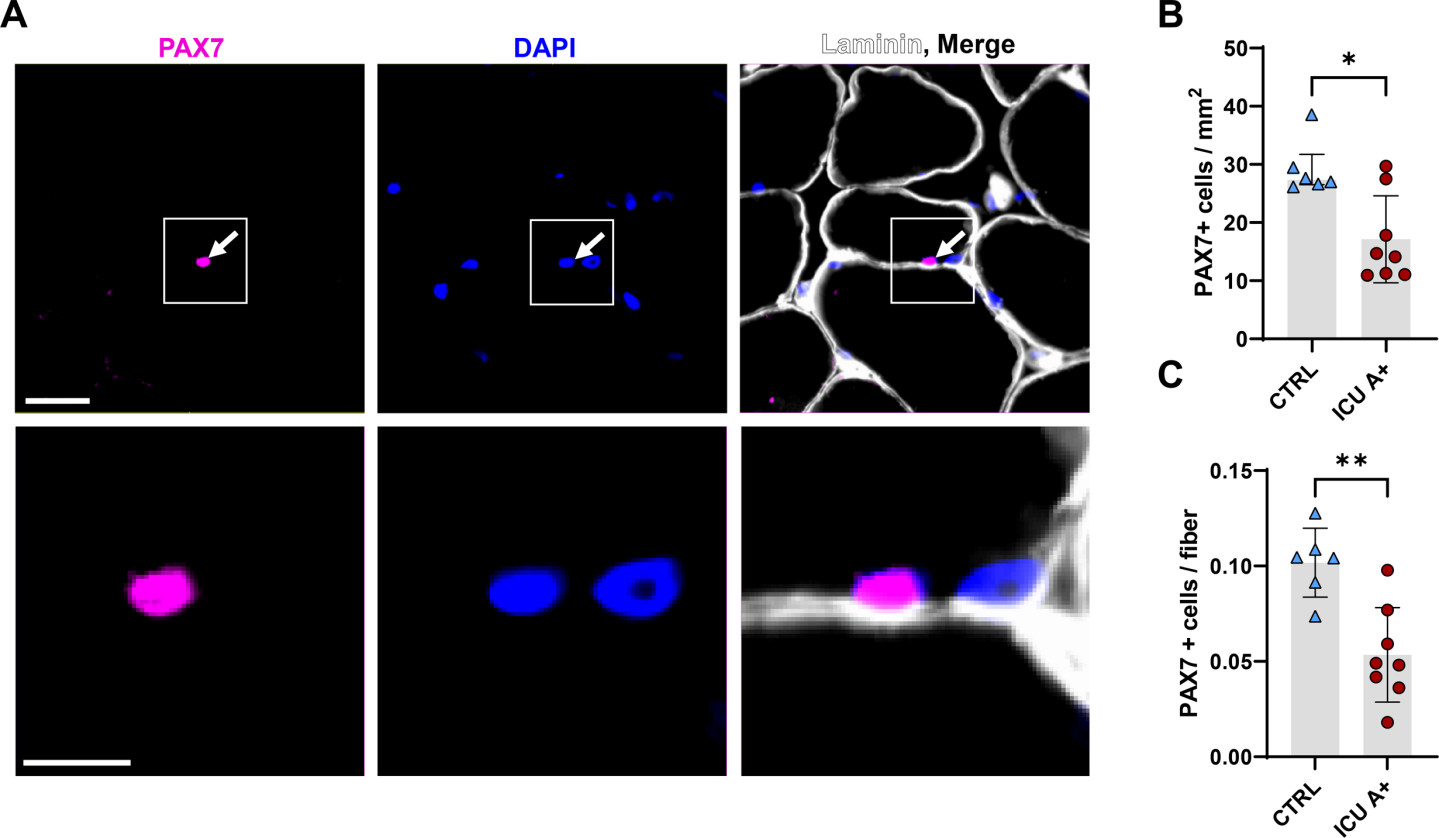
Decreased abundance of PAX7 positive cells in the atrophic diaphragm. (A) Representative images of diaphragm muscle cross‐sections stained with PAX7 antibody, Laminin antibody and DAPI. Nuclei with a PAX7‐positive signal were designated as satellite cells. Scale bar = 50 μm in the top row and 10 μm in the bottom row. (B) Number of PAX7 positive cells present in diaphragm muscle cross sections, normalized for section size. The grey bar represents the median [IQR] within the groups of patients. Each coloured symbol represents a single patient. CTRL *N* = 6; ICU *N* = 8. Significance level was calculated using Mann–Whitney U test. (C) Number of PAX7 positive cells present in diaphragm muscle cross sections, normalized for the number of myofibers. The grey bar represents the median [IQR] within the groups of patients, brackets represent interquartile ranges. Every coloured symbol represents a single patient. CTRL *N* = 6; ICU *N* = 8. Significance level was calculated using a Mann–Whitney U test. ICU = ICU group with atrophy, CTRL = Control group, * = *p* < 0.05, ** = *p* < 0.01.

After muscles undergo atrophy that is accompanied by loss of myonuclei (Figure 3) and transcriptional capacity (Figure 5), myonuclear number could be restored by the activation of satellite cells, the resident stem cells present adjacent to myofibers that contribute to myofiber homeostasis [14]. Thus, we aimed to investigate whether the muscle stem cell population in the atrophic diaphragm is affected, as this could further impair muscle recovery after atrophy. First, in our RNA‐seq data set, we found a lower relative expression of *PAX7*, a transcript uniformly expressed in muscle stem cells [15], in both ICU groups compared to the control group (Figure S1C). Next, immunofluorescent labeling of PAX7 was performed to determine the number of muscle stem cells in diaphragm cross‐sections (*N* = 6 Controls, *N* = 8 ICU patients) (Figure 6A). Patient characteristics of the groups used in these experiments are shown in Table S10. In ICU patients with atrophy, satellite cell content was reduced when normalized for either the number of myofibers present within the section (Figure 6B) or when normalized for total section area (Figure 6C). In conclusion, satellite cell content is reduced in the atrophic diaphragm of mechanically ventilated patients, potentially impeding the restoration of myonuclear number during recovery.

## 4 Discussion

In this study, we identified apoptotic pathway activation as a mechanism underlying critical illness‐associated diaphragm weakness. Furthermore, this is the first study to reveal reduced myonuclear number in mechanically ventilated ICU patients. We show that myonuclear loss is associated with reduced transcriptional activity within myofibers. Having lost a significant number of myonuclei may put patients at a disadvantage during weaning and may have long‐term consequences after ICU discharge, because muscle growth and recovery is associated with myonuclear number [10]. Reduced myonuclear number in atrophic myofibers implies that recovery of strength requires the addition of new myonuclei from satellite cells, instead of solely increasing protein synthesis to recover lost contractile material [16]. Furthermore, we discovered a reduced number of satellite cells in the diaphragm of ICU patients, possibly leading to further impairment of recovery after atrophy. Importantly, we did not find any relationship between diaphragm inactivity and myonuclear apoptosis, indicating that other factors such as inflammation and catabolism may play a more important role in ICU‐acquired diaphragm weakness.

### 4.1 Myonuclear Apoptosis in the Diaphragm

The fate of myonuclei in muscles undergoing atrophy is subject to debate [17]. The changes in myonuclear number and domain during atrophy likely depend on the type of atrophic stimulus [18, 19, 20]. Our observation that critical illness may induce caspase‐3‐mediated apoptosis of myonuclei is in accordance with the findings of a study investigating diaphragm atrophy in mechanically ventilated rats (6 and 12 h) [21]. The authors reported no changes in myonuclear domain size, indicating atrophy that was proportional to myonuclear loss. In our study, the myonuclear domain was significantly smaller in the ICU group with established atrophy compared to controls, implying more or faster atrophy than myonuclear loss. There are several potential explanations for this discrepancy. First, we used 3D rendering of Z‐stacks of confocal images to measure myonuclear domain volume within single myofibers, a more sensitive method compared to the myonuclear domain surface area (2D) measurements done on muscle cross‐sections in the rodent study [6]. Second, the patients in this study had a median ventilation duration of more than 60 h, much longer than the 12 h in the rodent study. In larger clinical studies, duration of ventilation was associated with the degree of diaphragm atrophy as measured by ultrasound, even though thickness measurements by ultrasound do not necessarily correlate with diaphragm myofiber thickness due to changes in the volume of the extracellular matrix in ICU patients [22]. The longer time on mechanical ventilation in our study possibly explains the reduction in myonuclear domain size, with atrophy exceeding myonuclear loss. Most importantly, unlike our critically ill patients, the mechanically ventilated rodents were healthy. Common conditions in the ICU such as systemic inflammation, increased metabolic demand, metabolic stress and drug exposure may independently cause muscle wasting, further accelerating atrophy [23]. The pathophysiology of diaphragm atrophy has also been evaluated in mechanically ventilated brain‐dead organ donors [24]. Oxidative stress and mitochondrial dysfunction were suggested to activate the intrinsic pathway leading to caspase‐3 activation and apoptosis [9]. In diaphragm biopsies of mechanically ventilated ICU patients, a redox imbalance was proposed to underlie diaphragm atrophy in the absence of mitochondrial dysfunction or oxidative stress. Again, this discrepancy may be due to the difference in clinical features between brain‐dead organ donors and ventilated critically ill patients. In quadriceps muscle biopsies of ICU patients, caspase‐3 activity was increased, albeit at a lower level than in the diaphragm, indicating caspase‐3 activation before the onset of muscle atrophy and loss of myonuclei, independent of mechanical ventilation. Indeed, in the quadriceps of mechanically ventilated ICU patients, atrophy has been shown to occur later (7 days) after ICU admission [25]. Furthermore, our findings may be explained by the longer duration of mechanical ventilation in the diaphragm cohort (Table S6). In our study, we found an increased apoptotic index of ‘other nuclei’ measured with activated caspase‐3 staining (Figure S3A,B), while the TUNEL‐based apoptotic index was not different between the groups. This discrepancy may be due to a shorter time window in which double‐stranded DNA‐breaks (that are made visible with TUNEL) are present compared to the time activated caspase‐3 is present in and around an apoptotic nucleus, as caspase‐3 activation occurs before the onset of apoptosis [26].

We report a transcriptomic profile of the diaphragm of mechanically ventilated ICU patients. Main findings include the upregulation of 20 genes associated with the p53 pathway, as well as the upregulation of 16 genes associated with apoptosis. Because p53 is subject to redox regulation, a redox imbalance may activate the pathway [27]. The p53 pathway was upregulated in various animal models of muscle atrophy. In mouse soleus muscle, after 48 h of hind limb suspension, the p53 pathway was upregulated and the apoptotic index was increased [28]. In a recent study in mechanically ventilated rabbits, the p53 pathway was upregulated and was hypothesized to contribute to diaphragm weakness via increased senescence [29]. Interestingly, the p53 and apoptosis pathways were not upregulated in a transcriptomics study of the atrophic diaphragm of controlled mechanically ventilated rats [30]. This demonstrates the disparity between animal models and clinical reality, further underlining the need to study pathophysiology in samples from critically ill patients. Finally, 19 genes associated with the integrin pathway were upregulated in the diaphragm of ICU patients. This pathway is associated with the transmission of forces from the extracellular matrix to actin and has been shown to be upregulated after eccentric (lengthening) contractions [31]. Eccentric contractions of the diaphragm have been hypothesized to occur in ventilated patients [32, 33]. Therefore, our data may support a role for eccentric contractions in the pathophysiology of critical illness associated diaphragm weakness.

In the RNA‐sequencing data presented in this study, proteolytic pathways were not significantly upregulated and protein synthesis pathways were not inhibited. This is surprising, as at the protein level, proteolytic pathways were upregulated in a previous study by our group performed in a similar cohort of patients [34]. One possibility is that these processes are not regulated at the transcript level.

### 4.2 Decreased Number of Satellite Cells in the Mechanically Ventilated Diaphragm

Our results show that the number of *PAX7* expressing cells is reduced in the diaphragm of mechanically ventilated ICU patients. This may be a direct effect of increased apoptosis, but we did not perform a separate experiment to quantify apoptotic satellite cells. Only a small fraction of cells in our cross‐sections expressed *PAX7* (0.05%–0.1%) Therefore, it is technically challenging to capture apoptotic satellite cells, especially because of diaphragm biopsy size limitations. Whether satellite cells are required for muscle regeneration depends on the type of atrophy and on whether myonuclei are lost during atrophy [14, 35, 36]. Nevertheless, lineage tracing studies demonstrated that satellite cell activity is particularly high in the diaphragm under non‐diseased conditions [37]. In addition, there are interstitial stem cells that can contribute to myofibers that do not express *PAX7* [38] (Supporting Information S1: S5–S6). Future studies should determine the prevalence of non‐satellite cell myogenic progenitors in the diaphragm of humans and whether these cells are a significant source of myonuclei during diaphragm muscle homeostasis. Satellite cell content was reduced in the quadriceps of mechanically ventilated patients with sustained atrophy after 6 months, suggesting a crucial role for satellite cells during muscle regeneration in ICU patients [39]. However, the exact role of satellite cells during recovery after diaphragm atrophy warrants further investigation.

Disturbed sarcomeric integrity is accepted as one of the mechanisms contributing to critical illness‐associated diaphragm weakness [34]. In non‐diseased conditions, muscle can repair its sarcomeres as damage occurs during both exercise and normal use. After sarcomeres are damaged, nuclear movement to a site of injury is required for repair [40]. During this process, myonuclei migrate toward damaged muscle sections to supply mRNA for sarcomeric proteins, allowing the reassembly of the contractile machinery [40]. This mechanism may be disturbed in critical illness‐associated diaphragm weakness because sarcomeric damage and reduced myonuclear number are present simultaneously.

### 4.3 Clinical Implications

The identification of intrinsic apoptotic pathway activation as a mechanism underlying diaphragm atrophy in mechanically ventilated ICU patients may open therapeutic venues to prevent and reverse diaphragm weakness and improve weaning success. The intrinsic apoptotic pathway plays a central role in many pathologies (Supporting Information S1: S7). Therefore, many inhibitors targeting the different components of this pathway have been developed (S8). Especially caspase‐3 inhibition has been investigated extensively and was shown to prevent diaphragm atrophy in mechanically ventilated rodents (Supporting Information S1: S8) [21]. To date, none have progressed beyond pre‐clinical studies, probably due to functions of caspase‐3 outside of the intrinsic apoptotic pathway (S9). However, there are other strategies to limit apoptosis, such as limiting redox imbalance or inhibiting other constituents of the intrinsic apoptotic pathway, such as BAX or BAK (Supporting Information S1: S8, S9). Additionally, increasing the generation of new myonuclei by stimulating satellite cell activation may be a strategy to help diaphragm recovery after atrophy and help the weaning process. Signalling pathway and immune modulators and various growth factors have been shown to augment satellite cell activation (Supporting Information S1: S10). More preclinical research is necessary to further explore these targets. Even though our data does not suggest that apoptotic pathway activation is limited to the diaphragm, we only observed myonuclear loss in the presence of myofiber atrophy, suggesting that these mechanisms are linked. Diaphragm unloading by the mechanical ventilator has been linked to diaphragm atrophy and weakness in numerous studies [24]. In our data, there was no relationship between diaphragm inactivity and myonuclear apoptosis, suggesting a more prominent role for other drivers of diaphragm weakness, such as inflammation or increased catabolism. Indeed, we found upregulation of cytokine‐cytokine receptor interaction in ICU patients with diaphragm atrophy compared to patients without atrophy. Blood markers of inflammation did not correlate with markers of apoptosis, suggesting that markers of systemic inflammation may not reflect the condition of the diaphragm. Recent studies investigating the effect of early diaphragm pacing to prevent unloading of the diaphragm during controlled ventilation did not decrease the duration of mechanical ventilation, suggesting that mechanisms other than diaphragm unloading are important (Supporting Information S1: S11, S12). Furthermore, in another recent study, diaphragm hibernation was shown to contribute to weakness and was not correlated with duration of mechanical ventilation [4]. Taken together, this supports the notion that ICU‐acquired diaphragm dysfunction has a complex underlying pathophysiology and cannot be explained by disuse atrophy only.

### 4.4 Limitations

The population of ICU patients included in this is highly heterogeneous, with varying medical histories, reasons for admission, duration of mechanical ventilation, use of medication that influences muscle function and underlying pathophysiology. Therefore, it is challenging to search for clinical predictors associated with myonuclear apoptosis or myofiber atrophy with our sample size. Even though chronic use of corticosteroids was an exclusion criterium, the use of medications in the ICU may have impacted the results presented in this study, as corticosteroids and neuromuscular blockers increase the risk of diaphragm dysfunction and probably, atrophy [23]. Nevertheless, this diverse group of patients does adequately reflect the general ICU population. In this study, we were able to acquire diaphragm biopsies when mechanically ventilated ICU patients underwent surgery for a clinical reason. Therefore, we could not time the moment of biopsy and we were not able to perform longitudinal measurements of diaphragm function before biopsies were obtained. Unfortunately, it was not feasible to obtain multiple biopsies (i.e., pre‐ and post‐ICU admission). The absence of time‐course data is a major limitation of the study. Diaphragm atrophy was not assessed in the cohort of patients that underwent a quadriceps biopsy due to the invasiveness of the biopsies. Additionally, the number of control quadriceps biopsies was relatively low (*N* = 5). This limits the conclusions that can be drawn based on the experiments performed on these biopsies. Due to the invasiveness of taking diaphragm biopsies, the controls in this study are patients undergoing surgery for a pulmonary nodule. We cannot rule out an effect of the clinical status of the controls on the diaphragm, even though the inclusion and‐exclusion criteria should minimize this. Finally, due to biopsy size limitations, not all experiments were performed on all biopsies.

## 5 Conclusion

In mechanically ventilated patients in the ICU, myonuclear apoptosis is a pathophysiological mechanism underlying critical illness‐associated diaphragm atrophy. Using a combination of advanced microscopy and molecular biology techniques, we identified p53 activation as the underlying pathway in ICU patients. Unloading of the diaphragm by the ventilator likely does not drive this mechanism, because there was no relationship between duration or mode of mechanical ventilation and apoptosis. Myonuclear loss in combination with a reduction of the satellite cell population may compromise recovery of the diaphragm, thereby contributing to weaning failure.

## Funding

Supported by NHLBI grant HL‐121500 (C. A.C.O.); ZonMW Grant 09120011910004 (C.A.C.O.; L.H.); NIAMS R01AR084850 (T.J.K).

## Conflicts of Interest

The authors declare no conflicts of interest.

## Supporting information


**Figure S1:** Relative expression levels of genes associated with the P53 pathway.
**Figure S2:** Representative images of DNAse‐treated muscle cross‐section after TUNEL staining.
**Figure S3:** Apoptotic index of non‐myonuclei.
**Figure S4:** Increased Caspase‐3 index in Quadriceps biopsies of mechanically ventilated patients.
**Figure S4:** Increased Caspase‐3 index in Quadriceps biopsies of MV patients.
**Figure S5:** Proportion of PCM‐1 + nuclei within manually isolated single myofibers.
**Figure S6:** Myofiber volume, myonuclear number and myonuclear domain of isolated myofibers in two ICU groups.
**Figure S7:** Myonuclear content of diaphragm and quadriceps cross‐sections.
**Figure S6:** Myofiber volume, myonuclear number and myonuclear domain of isolated diaphragm myofibers in ICU patients and controls.
**Figure S7:** Myonuclear content of diaphragm and quadriceps cross‐sections.
**Figure S8:** Correlations of duration of mechanical ventilation and duration of diaphragm inactivity with myonuclear number and TUNEL, caspase‐3 Indices.
**Figure S9:** Correlations of respiratory outcomes with myonuclear number of myofibers.
**Figure S10:** Relative expression levels of genes associated with the P53 pathway in ICU patients with and without atrophy.
**Figure S11:** Correlations of plasma C‐reactive protein and leucocyte counts with myonuclear number of myofibers and apoptotic indices.
**Figure S12:** Scatterplots of nuclear number vs. myofiber cross‐sectional area and myonuclear domain vs. cross‐sectional area.

**Table S1:** Experiments performed per diaphragm biopsy.
**Table S2:** Patient characteristics, RNA‐sequencing experiment.
**Table S3:** Patient characteristics, TUNEL experiment.
**Table S4:** Patient characteristics, Caspase‐3 staining experiment.
**Table S5:** Patient characteristics, quadriceps experiment.
**Table S6:** Patient characteristics, diaphragm cohort compared to quadriceps cohort.
**Table S7:** PCM‐1 staining of manually isolated myofibers.
**Table S8:** Additional clinical characteristics of ICU patients with and without atrophy.
**Table S9:** Patient characteristics, transcriptional activity of nuclei experiment.
**Table S10:** Patient characteristics, PAX‐7 staining experiment.
**Table S12:** Composition of solutions.
**Table S13:** Primary antibodies.
**Table S14:** Secondary antibodies.
