# Supplementary material for: Loss of Myonuclei and Transcriptional Activity During Diaphragm Atrophy in Critically Ill Patients
Source: J Cachexia Sarcopenia Muscle. 2026 Feb 15;17(1):e70228. doi: 10.1002/jcsm.70228 (PMC12907519; doi:10.1002/jcsm.70228)
Supplement: Supplementary file 1 — Figure S1: Relative expression levels of genes associated with the P53 pathway. Figure S2: Representative images of DNAse‐treated muscle cross‐section after TUNEL staining. Figure S3: Apoptotic index of non‐myonuclei. Figure S4: Increased Caspase‐3 index in Quadriceps biopsies of mechanically ventilated patients. Figure S4: Increased Caspase‐3 index in Quadriceps biopsies of MV patients. Figure S5: Proportion of PCM‐1 + nuclei within manually isolated single myofibers. Figure S6: Myofiber volume, myonuclear number and myonuclear domain of isolated myofibers in two ICU groups. Figure S7: Myonuclear content of diaphragm and quadriceps cross‐sections. Figure S6: Myofiber volume, myonuclear number and myonuclear domain of isolated diaphragm myofibers in ICU patients and controls. Figure S7: Myonuclear content of diaphragm and quadriceps cross‐sections. Figure S8: Correlations of duration of mechanical ventilation and duration of diaphragm inactivity with myonuclear number and TUNEL, caspase‐3 Indices. Figure S9: Correlations of respiratory outcomes with myonuclear number of myofibers. Figure S10: Relative expression levels of genes associated with the P53 pathway in ICU patients with and without atrophy. Figure S11: Correlations of plasma C‐reactive protein and leucocyte counts with myonuclear number of myofibers and apoptotic indices. Figure S12: Scatterplots of nuclear number vs. myofiber cross‐sectional area and myonuclear domain vs. cross‐sectional area. [file JCSM-17-e70228-s001.pdf]

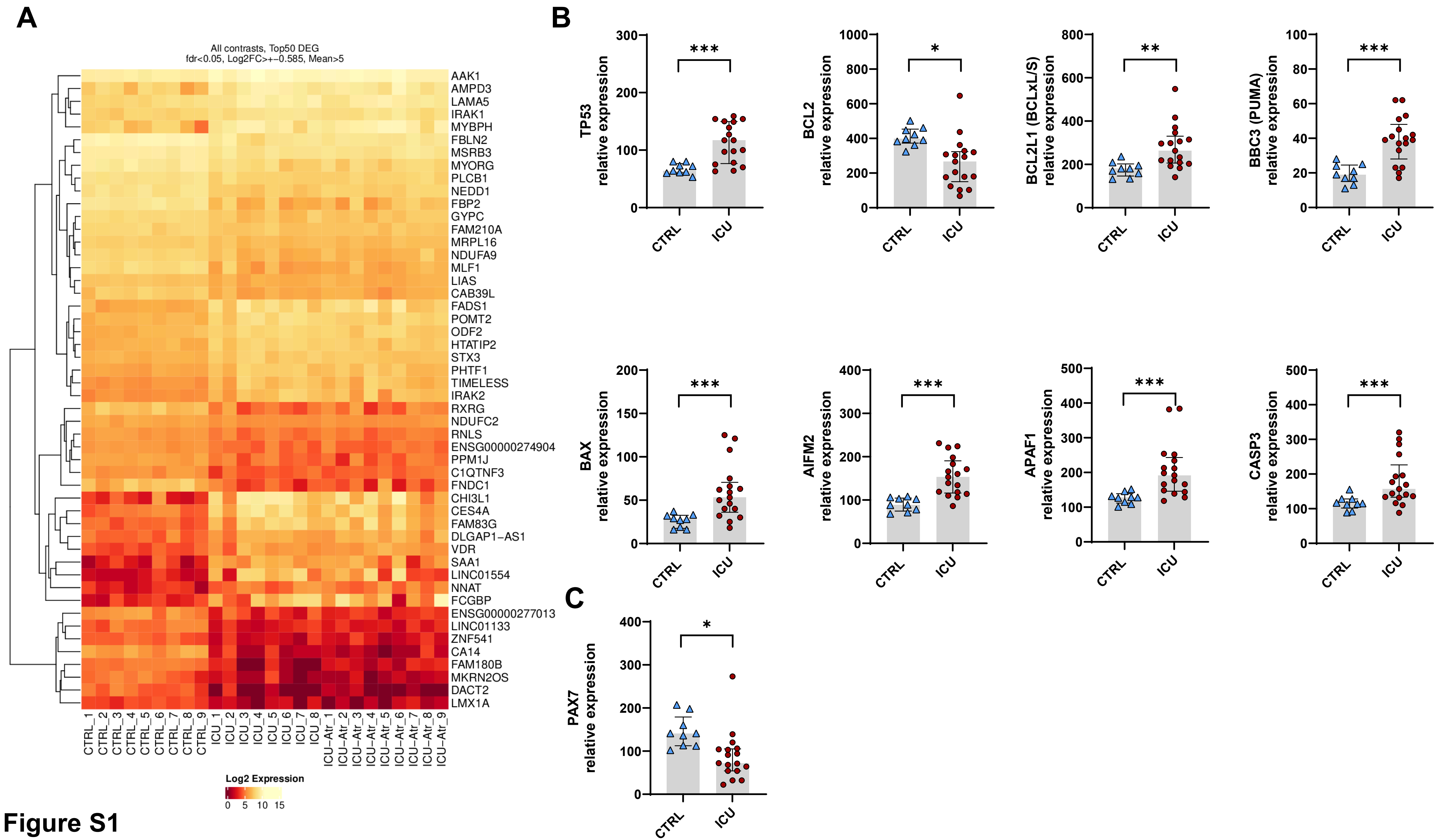
A
All contrasts, Top50 DEG
fdr<0.05, Log2FC>+-0.585, Mean>5
AAK1
AMPD3
LAMA5
IRAK1
MYBPH
FBLN2
MSRB3
MYORG
PLCB1
NEDD1
FBP2
GYPC
FAM210A
MRPL16
NDUFA9
MLF1
LIAS
CAB39L
FADS1
POMT2
ODF2
HTATIP2
STX3
PHTF1
TIMELESS
IRAK2
RXRG
NDUFC2
RNLS
ENSG00000274904
PPM1J
C1QTNF3
FNDC1
CHI3L1
CES4A
FAM83G
DLGAP1-AS1
VDR
SAA1
LINC01554
NNAT
FCGBP
ENSG00000277013
LINC01133
ZNF541
CA14
FAM180B
MKRN2OS
DACT2
LMX1A
CTRL_1 CTRL_2 CTRL_3 CTRL_4 CTRL_5 CTRL_6 CTRL_7 CTRL_8 CTRL_9
ICU_1 ICU_2 ICU_3 ICU_4 ICU_5 ICU_6 ICU_7 ICU_8
ICU-Atr_1 ICU-Atr_2 ICU-Atr_3 ICU-Atr_4 ICU-Atr_5 ICU-Atr_6 ICU-Atr_7 ICU-Atr_8 ICU-Atr_9
Log2 Expression
0 5 10 15
B
TP53 relative expression
BCL2 relative expression
BCL2L1 (BCLxL/S) relative expression
BBC3 (PUMA) relative expression
BAX relative expression
AIFM2 relative expression
APAF1 relative expression
CASP3 relative expression
CTRL ICU
***
*
**
C
PAX7 relative expression

Figure S1

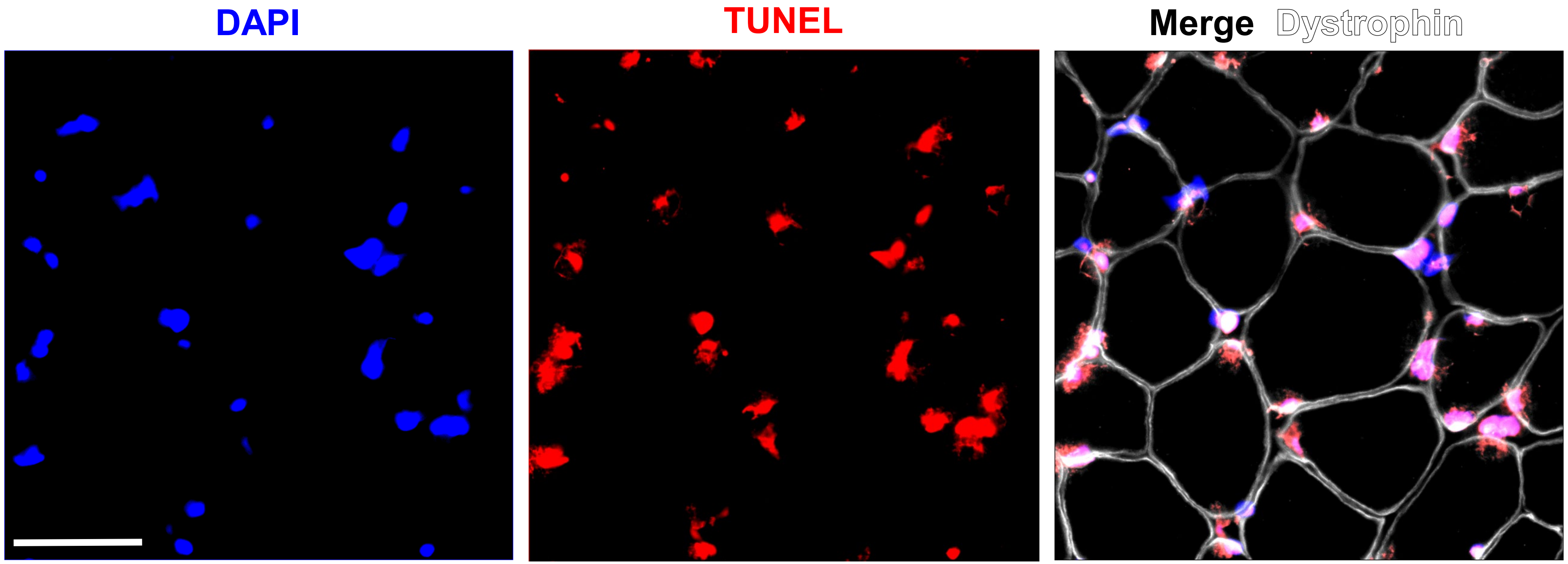

Figure S2

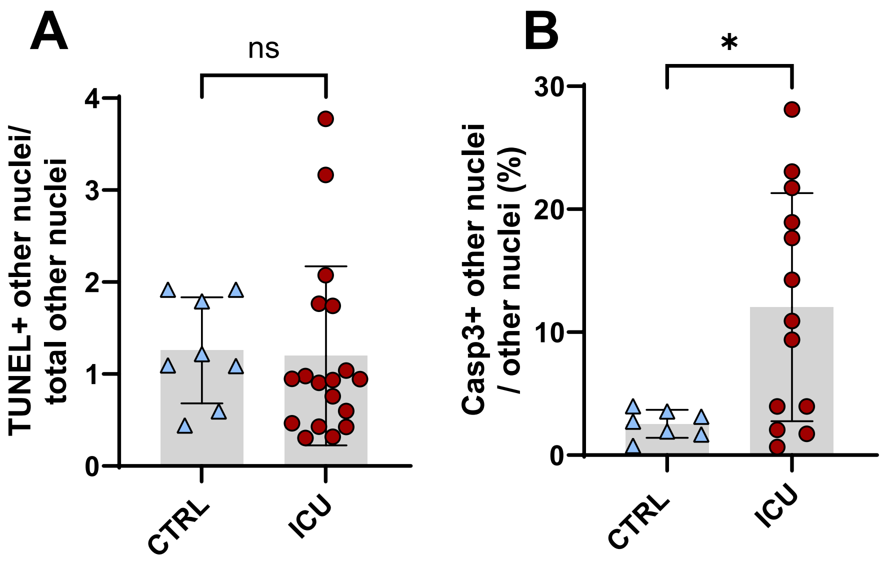
A
ns
TUNEL+ other nuclei/
total other nuclei
0
1
2
3
4
CTRL
ICU
B
*
Casp3+ other nuclei
/ other nuclei (%)
0
10
20
30
CTRL
ICU

Figure S3

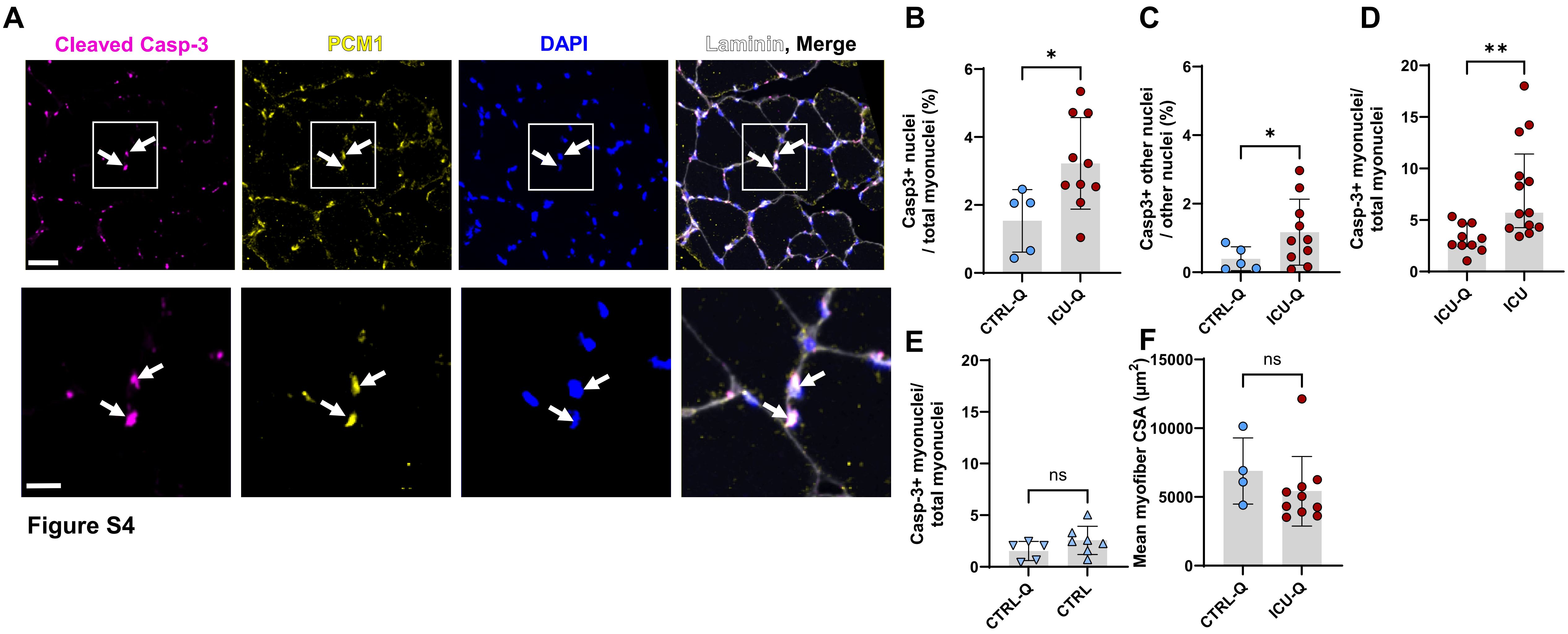
A
Cleaved Casp-3
PCM1
DAPI
Laminin, Merge
B
Casp3+ nuclei / total myonuclei (%)
*
CTRL-Q
ICU-Q
C
Casp3+ other nuclei / other nuclei (%)
*
CTRL-Q
ICU-Q
D
Casp-3+ myonuclei/ total myonuclei
**
ICU-Q
ICU
E
Casp-3+ myonuclei/ total myonuclei
ns
CTRL-Q
CTRL
F
Mean myofiber CSA (μm²)
ns
CTRL-Q
ICU-Q

Figure S4

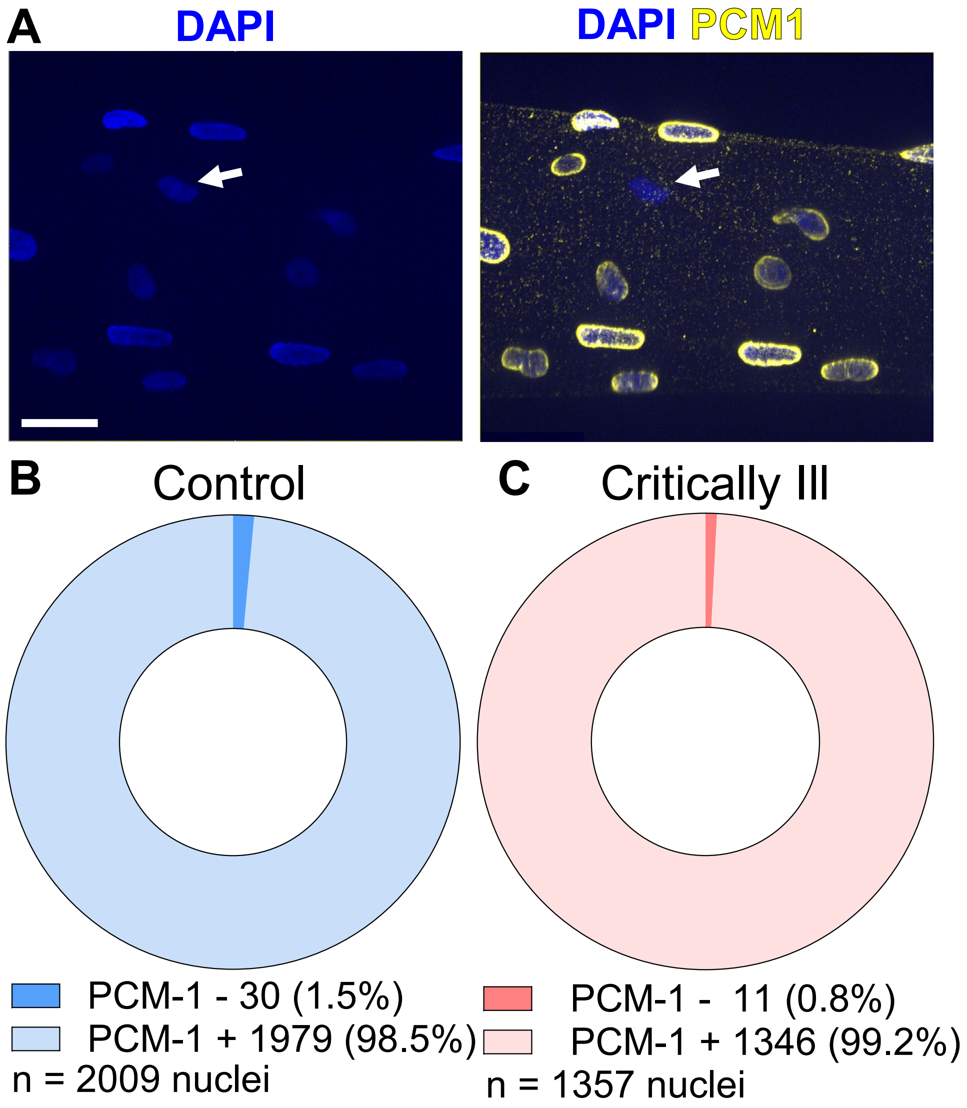
A
DAPI
DAPI PCM1
B
Control
C
Critically Ill
PCM-1 - 30 (1.5%)
PCM-1 + 1979 (98.5%)
n = 2009 nuclei
PCM-1 - 11 (0.8%)
PCM-1 + 1346 (99.2%)
n = 1357 nuclei

Figure S5

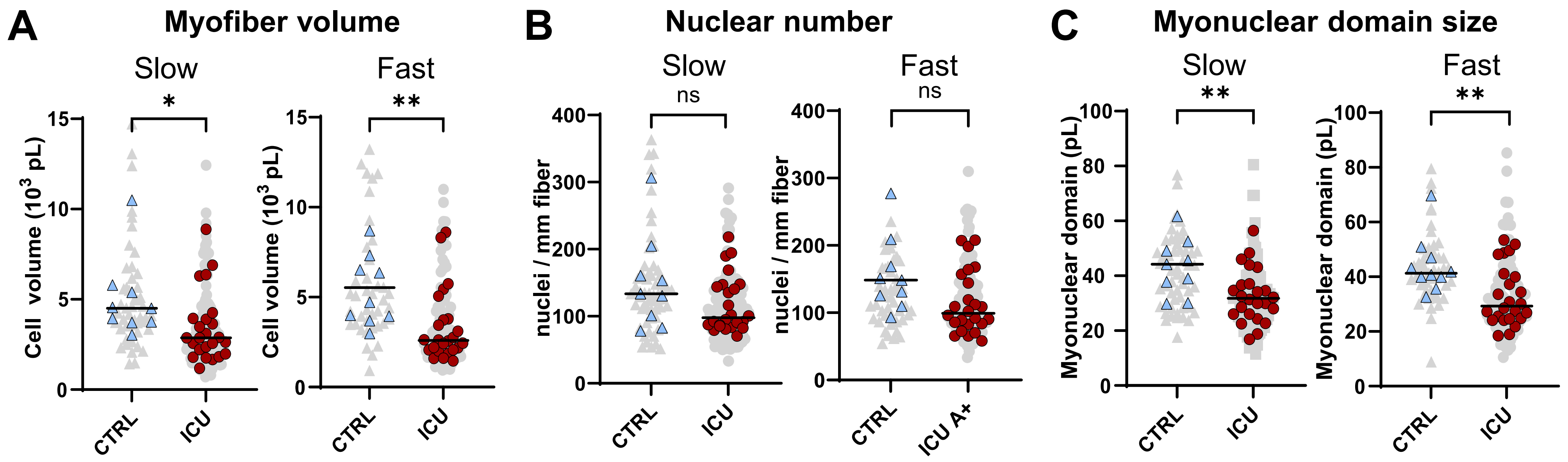
A
Myofiber volume
Slow
Fast
*
**
Cell volume (10³ pL)
15
10
5
0
CTRL
ICU
B
Nuclear number
Slow
Fast
ns
nuclei / mm fiber
400
300
200
100
0
CTRL
ICU
ICU A+
C
Myonuclear domain size
Slow
Fast
**
Myonuclear domain (pL)
100
80
60
40
20
0
CTRL
ICU

Figure S6

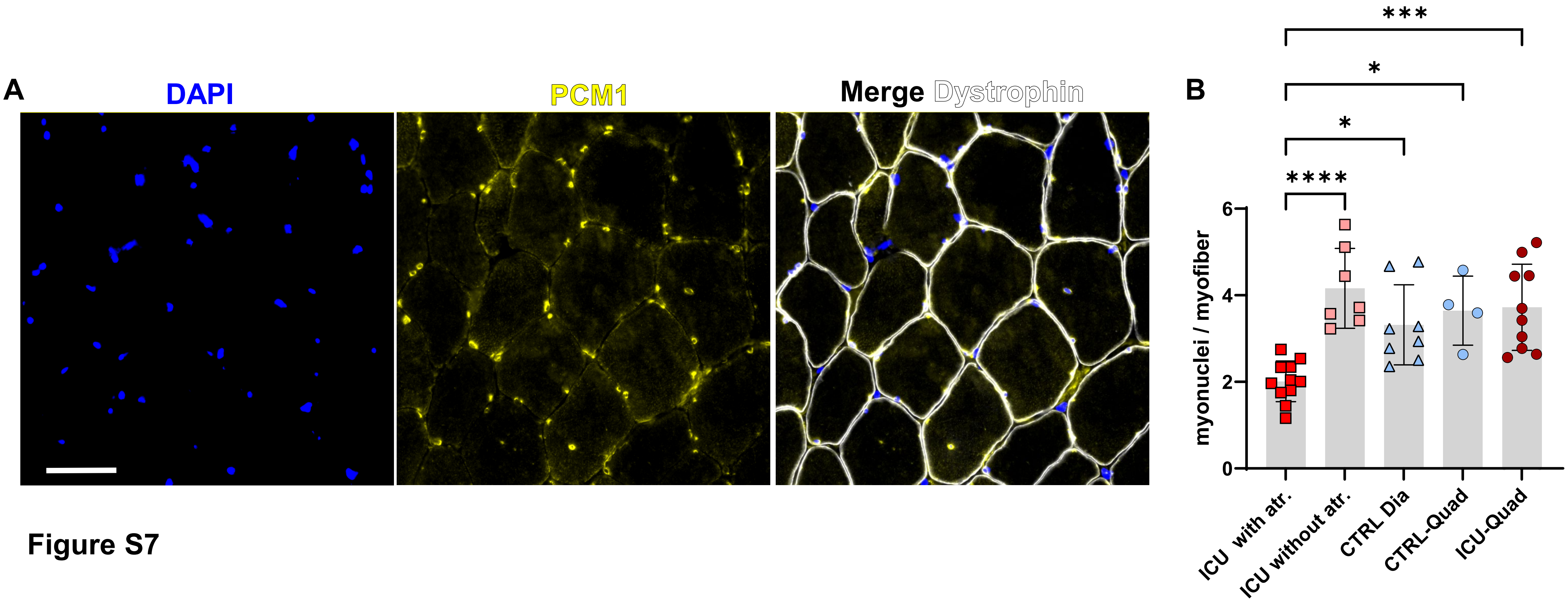
A
DAPI
PCM1
Merge Dystrophin
B
****
*
*
***
myonuclei / myofiber
0
2
4
6
ICU with atr.
ICU without atr.
CTRL Dia
CTRL-Quad
ICU-Quad

Figure S7

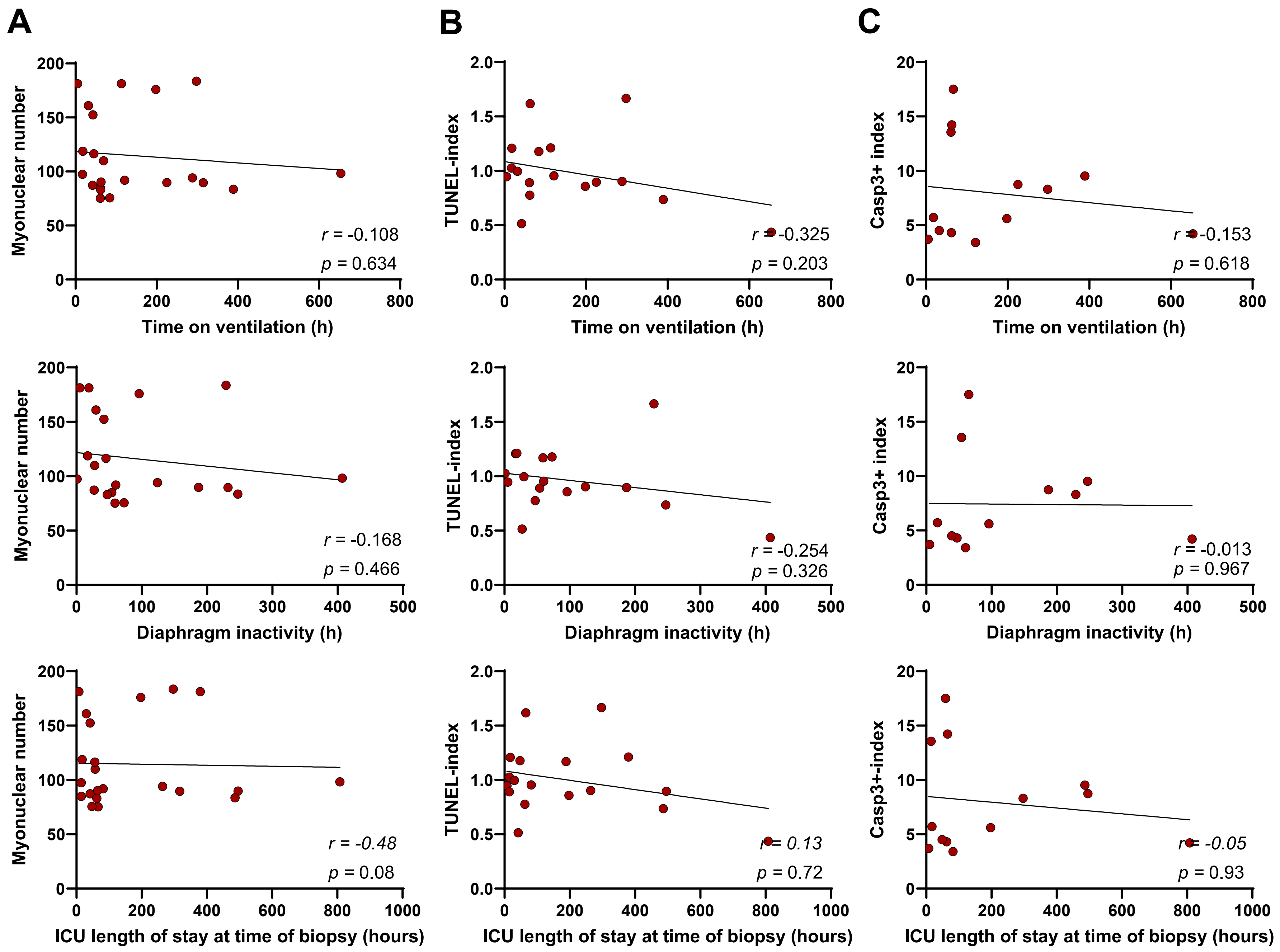
A
B
C
Myonuclear number
Time on ventilation (h)
r = -0.108
p = 0.634
TUNEL-index
r = -0.325
p = 0.203
Casp3+ index
r = -0.153
p = 0.618
Diaphragm inactivity (h)
r = -0.168
p = 0.466
r = -0.254
p = 0.326
r = -0.013
p = 0.967
ICU length of stay at time of biopsy (hours)
r = -0.48
p = 0.08
r = 0.13
p = 0.72
Casp3+-index
r = -0.05
p = 0.93

Figure S8

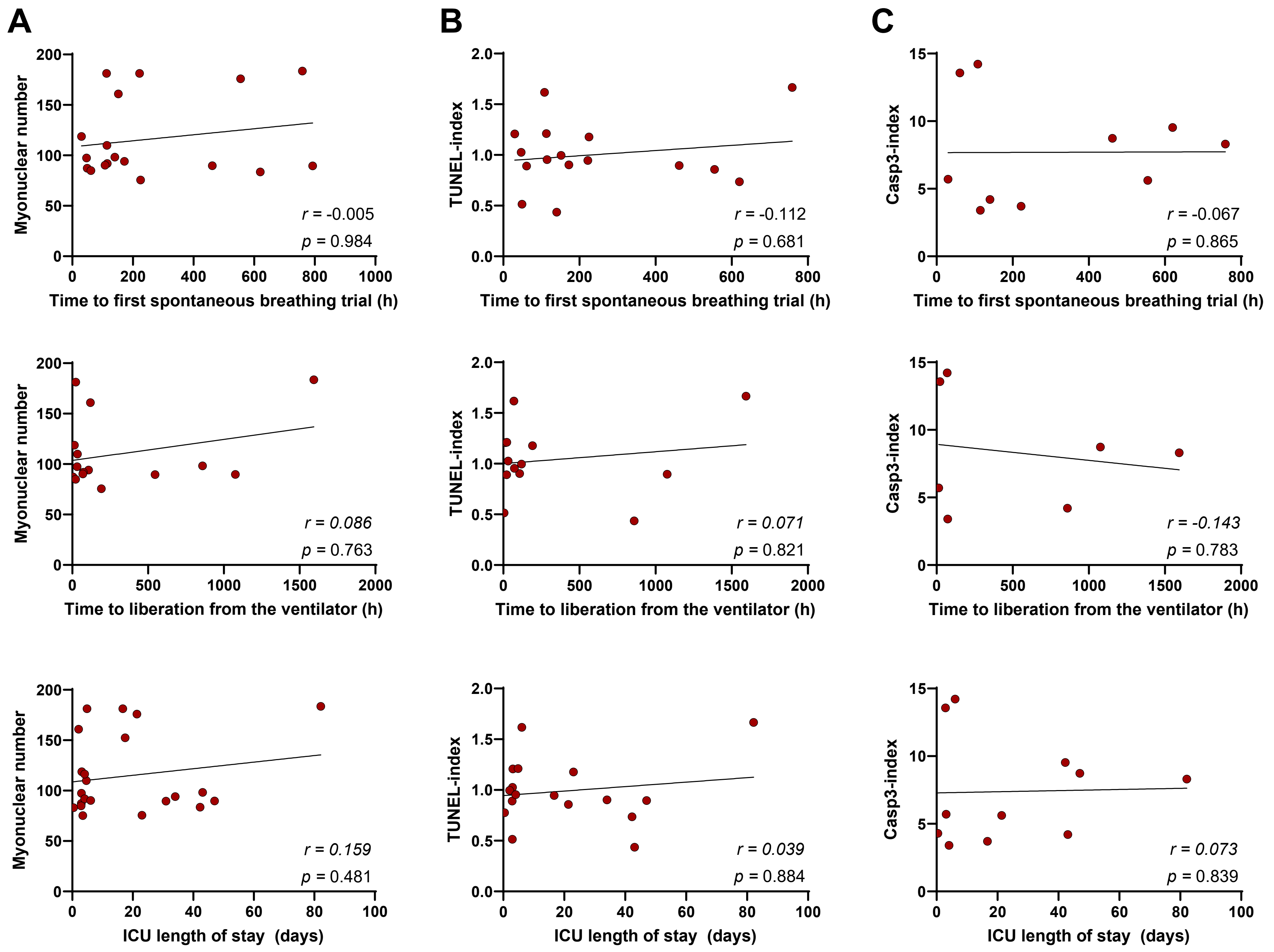
A
B
C
Myonuclear number
TUNEL-index
Casp3-index
Time to first spontaneous breathing trial (h)
Time to liberation from the ventilator (h)
ICU length of stay (days)
r = -0.005
p = 0.984
r = -0.112
p = 0.681
r = -0.067
p = 0.865
r = 0.086
p = 0.763
r = 0.071
p = 0.821
r = -0.143
p = 0.783
r = 0.159
p = 0.481
r = 0.039
p = 0.884
r = 0.073
p = 0.839

Figure S9

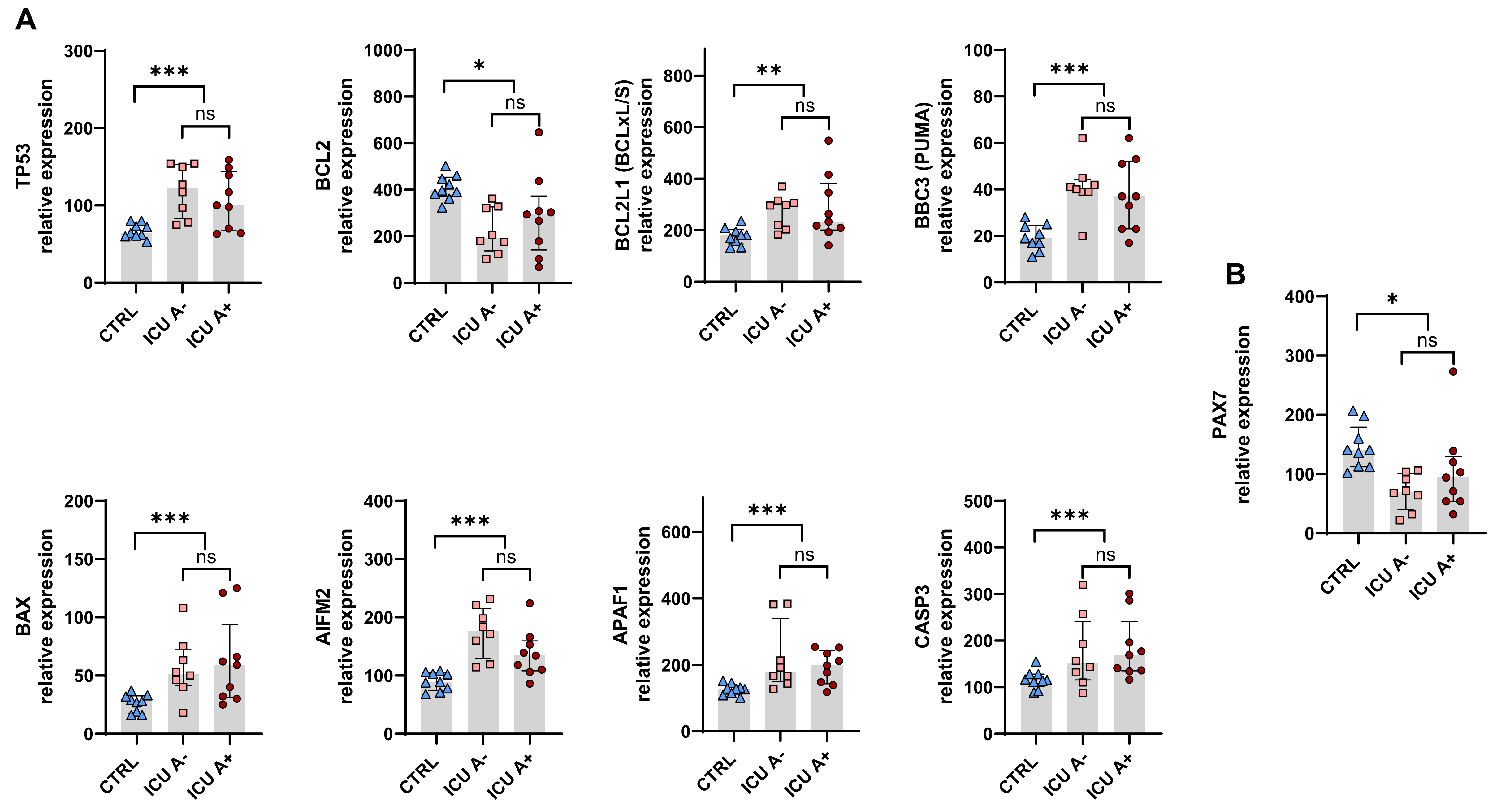
A
TP53 relative expression
BCL2 relative expression
BCL2L1 (BCLxL/S) relative expression
BBC3 (PUMA) relative expression
BAX relative expression
AIFM2 relative expression
APAF1 relative expression
CASP3 relative expression
B
PAX7 relative expression
CTRL
ICU A-
ICU A+
***
**
*
ns

Figure S10

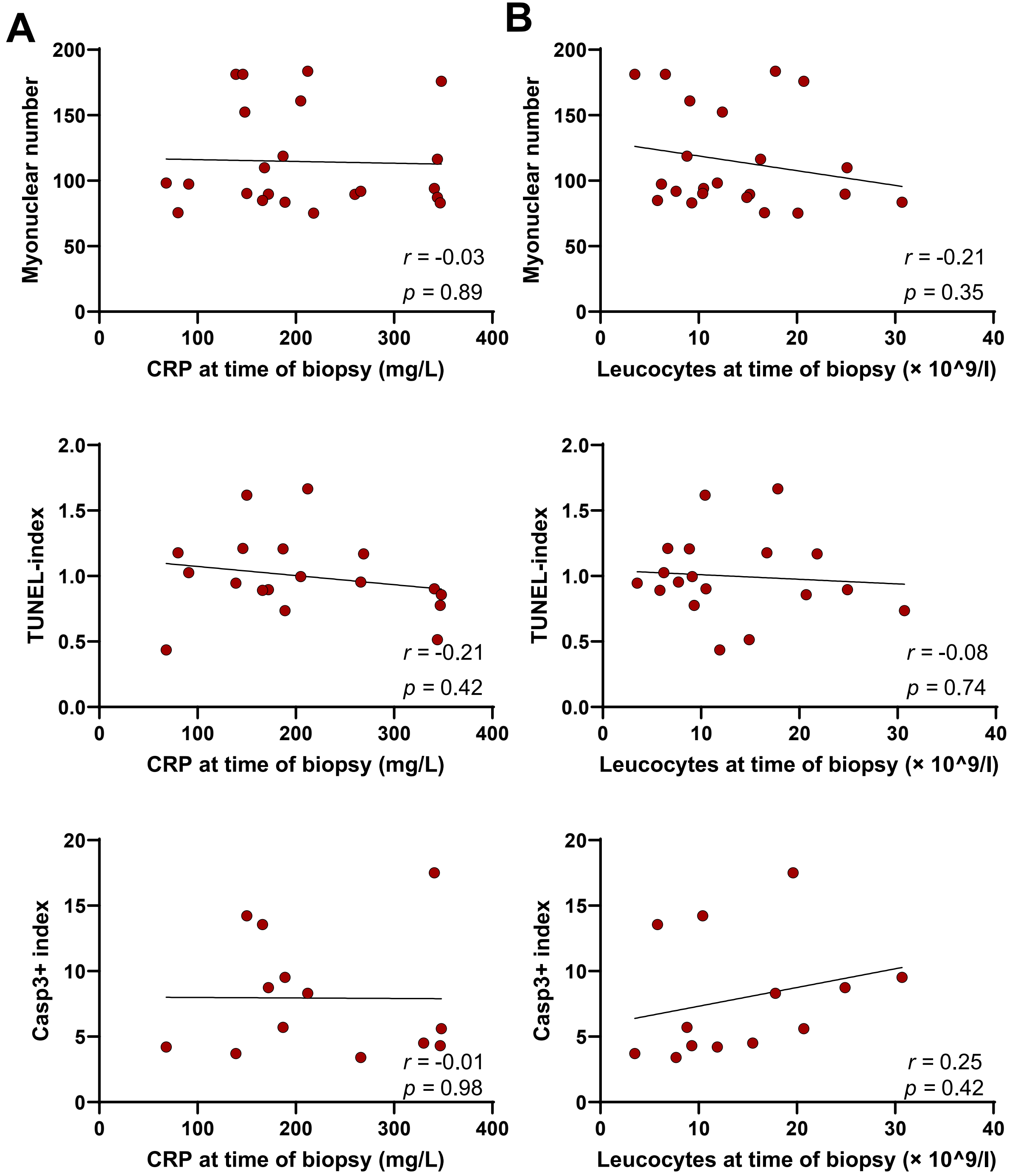
A
B
Myonuclear number
CRP at time of biopsy (mg/L)
r = -0.03
p = 0.89
Leucocytes at time of biopsy (× 10^9/l)
r = -0.21
p = 0.35
TUNEL-index
r = -0.21
p = 0.42
r = -0.08
p = 0.74
Casp3+ index
r = -0.01
p = 0.98
r = 0.25
p = 0.42

Figure S11

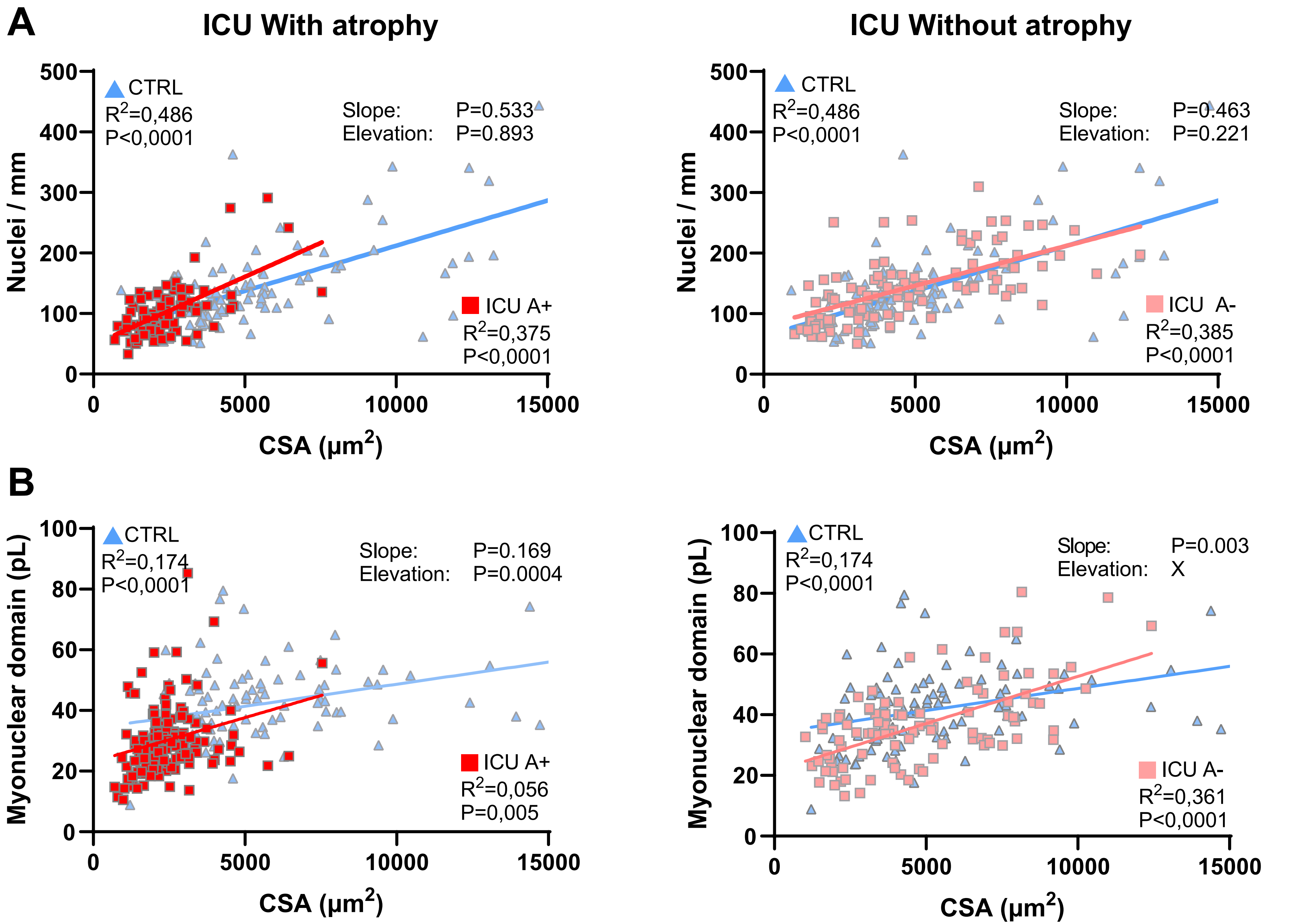
A
ICU With atrophy
ICU Without atrophy
CTRL
R2=0,486
P<0,0001
Slope: P=0.533
Elevation: P=0.893
ICU A+
R2=0,375
P<0,0001
Nuclei / mm
CSA (μm2)
Slope: P=0.463
Elevation: P=0.221
ICU A-
R2=0,385
P<0,0001
B
R2=0,174
Slope: P=0.169
Elevation: P=0.0004
R2=0,056
P=0,005
Myonuclear domain (pL)
Slope: P=0.003
Elevation: X
R2=0,361

Figure S12

Graphical summary

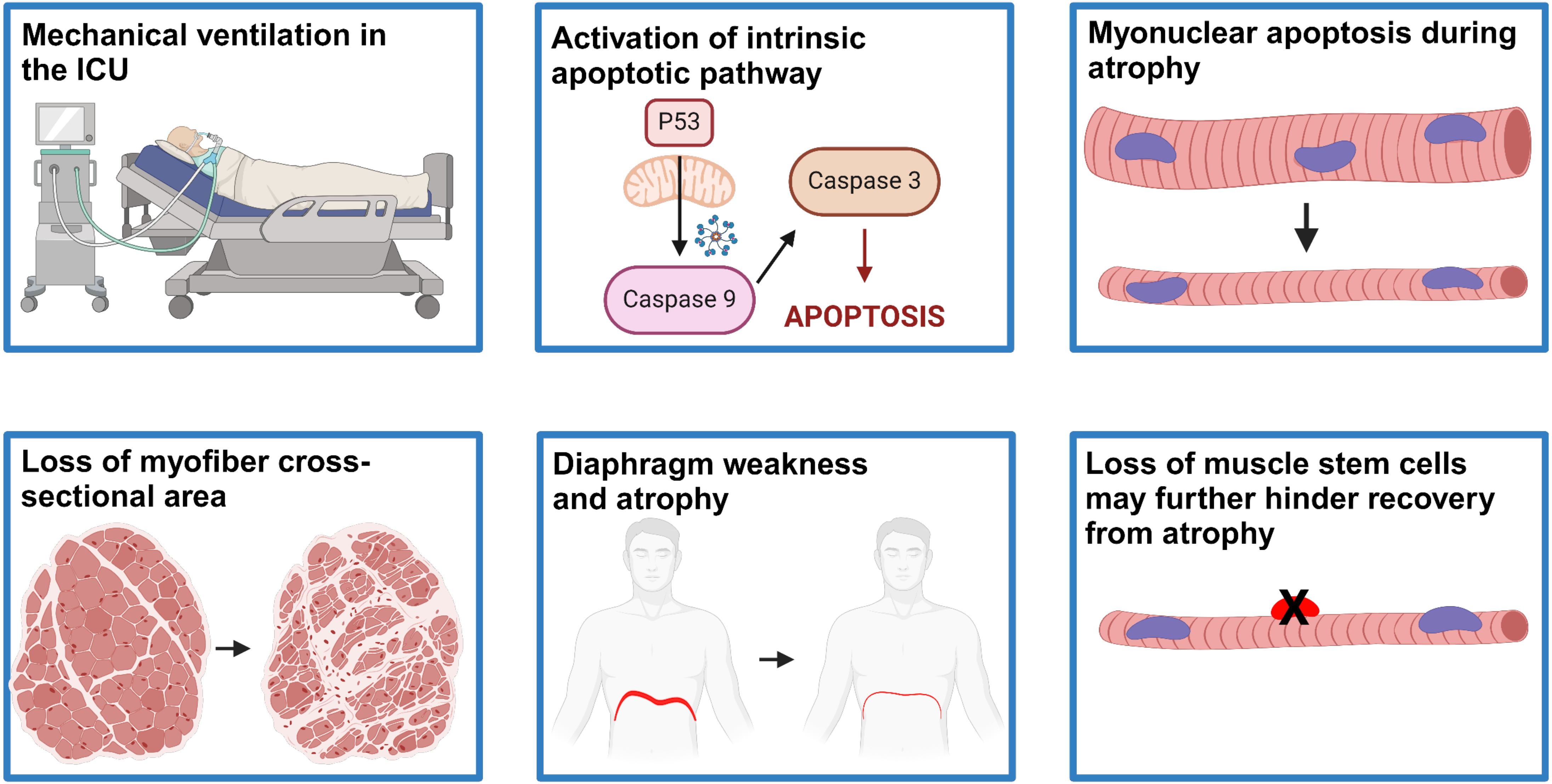

Figure S13
