# Supplementary material for: Loss of Myonuclei and Transcriptional Activity During Diaphragm Atrophy in Critically Ill Patients
Source: J Cachexia Sarcopenia Muscle. 2026 Feb 15;17(1):e70228. doi: 10.1002/jcsm.70228 (PMC12907519; doi:10.1002/jcsm.70228)
Supplement: Supplementary file 2 — Table S1: Experiments performed per diaphragm biopsy. Table S2: Patient characteristics, RNA‐sequencing experiment. Table S3: Patient characteristics, TUNEL experiment. Table S4: Patient characteristics, Caspase‐3 staining experiment. Table S5: Patient characteristics, quadriceps experiment. Table S6: Patient characteristics, diaphragm cohort compared to quadriceps cohort. Table S7: PCM‐1 staining of manually isolated myofibers. Table S8: Additional clinical characteristics of ICU patients with and without atrophy. Table S9: Patient characteristics, transcriptional activity of nuclei experiment. Table S10: Patient characteristics, PAX‐7 staining experiment. Table S12: Composition of solutions. Table S13: Primary antibodies. Table S14: Secondary antibodies. [file JCSM-17-e70228-s002.docx]

**Online Supplement**

*Patients, Diaphragm studies*
In this multicenter study, diaphragm muscle biopsies were taken from intensive care unit (ICU) patients receiving invasive mechanical ventilation (*ICU* patients, n=24) and patients undergoing elective lung surgery for early-stage lung malignancy without critical illness (*Control* patients, n=10). Exclusion criteria were chronic obstructive pulmonary disease (≥ GOLD stage III), congestive heart failure, neuromuscular diseases, chronic metabolic disorders, pulmonary hypertension, chronic use of corticosteroids (> 7.5mg/day for at least 3 months), and more than 10% weight loss within the last 6 months. The exclusion criteria were similar for all experimental groups. Patients’ characteristics of the two groups are presented in Tables 1 and E1/E2. The biopsy protocol was approved by the institutional review board at Amsterdam UMC (location VUmc), the Netherlands. Patients were recruited in Amsterdam UMC and the Netherlands Cancer Institute-Antoni van Leeuwenhoek Hospital (both in Amsterdam, the Netherlands), and Medisch Spectrum Twente (Enschede, the Netherlands). Written informed consent was obtained from the patients or their legal representative. Biopsies were stored at -80⁰C.

*Patients, Quadriceps studies*

Quadriceps biopsies of ICU patients (*n* = 10) were obtained in the context of a separate study that has been filed in the Clinical Trial Register under #NCT03231540 and was approved by the Medical Ethical Committee of VU Medical Center, Amsterdam, the Netherlands. Informed consent was obtained from the patient or a legal representative. Patients ≥18 years that had an expected ventilation duration of ≥72 hours, were expected to tolerate enteral nutrition ≥72 hours, and had a Sequential Organ Failure Assessment (SOFA) score ≥6, were considered eligible for inclusion. Exclusion criteria were contra-indications to enteral nutrition, short bowel syndrome, type C liver cirrhosis or acute liver failure, dependency on renal replacement therapy, requiring another specific enteral nutrition formula for medical reasons, BMI >35 kg/m2, extensive treatment limitations, disseminated malignancy, hematological malignancy, primary neuromuscular pathology, chronic use of corticosteroids for >7 days before ICU admission or contra-indications for muscle biopsy such as the need for continuous systemic anticoagulation, prothrombin time >1.3 or thrombocytes <100.

*Histology, myofiber cross-sectional area*
We used a previously described method for muscle specimen handling (1). Cryosections (8 µm thick, perpendicular to myofiber direction) were cut from the frozen biopsies and stained to study 1) myofiber type using myosin heavy chain antibodies; fast MHC (Table S13, #1), 2) myofiber cross-sectional area using wheat germ agglutinin (WGA, 1:25 diluted in PBS-0.5% BSA, Molecular Probes) staining. Myofiber cross-sectional area was obtained from whole cryosections.

*Single muscle fibers: myofiber volume and nuclear count*
Diaphragm biopsies were cut while submerged in liquid nitrogen. Segments of single diaphragm myofibers (length: 1-1.5 mm) were isolated in a relaxing solution at 5ºC (solution composition in supplementary Table 3. Single myofibers were slightly stretched to improve antibody penetration and attached to custom-made aluminum clips that were glued to glass coverslips ~2 mm apart, 6 pairs of aluminum clips per slide, and 2 slides per biopsy. A hydrophobic barrier was drawn using a PAP pen. The myofibers were fixed in 4% PFA for 15 minutes and permeabilized for 15 minutes and 1% triton X-100 in PBS. After blocking with 1% BSA in PBS, the fibers were incubated overnight with primary antibodies staining for Lamin A/C, MHC pan and fast MHC (Table S13, #2-4), at 4 C. The fibers were incubated overnight at 4C with secondary antibodies (Table S14, #1, #2, #3). Next, slides were incubated with DAPI (2µ/mL) for 5 minutes at room temperature and the samples were mounted in Vectashield (Vector Labs) and imaged the following week. The fibers were imaged using a CrestOptics X3 spinning disk confocal microscope using a Zeiss 63x magnification objective. Z-step size was 5 um, and three z-stacks with a thickness of the fiber thickness and a length of 208.5 μm were imaged. The Z-stacks were loaded into Imaris (Oxford Instruments) analysis software. Myofibers and nuclei were segmented using automatic thresholding using the MHC and Lamin A/C staining after a background subtraction pre-processing step. Each Z-stack was checked for wrongfully segmented nuclei and adjusted accordingly. Sarcomere length was determined in each z-stack using the MyofibrilJ plugin in FIJI ([https://imagej.net/**MyofibrilJ**](https://imagej.net/MyofibrilJ)) on a single Z-slice of Myosin heavy chain staining. Nuclear counts were normalized for sarcomere length because the amount of myofiber that was present in the imaging window varies with myofiber stretch. Z-stacks of the Lamin A/C images were used to generate sum intensity projections using FIJI. The nuclei present in each fiber were segmented using the Stardist nuclear segmentation plugin for FIJI. Overlaying nuclei were excluded from the analysis. Wrinkling index of each nucleus was determined by calculating the standard deviation of the fluorescence intensity variation of Lamin A/C staining within the region of interest of each nucleus (2).

*Single muscle fibers: transcriptional activity*
The transcriptional activity of each nucleus was determined by measuring the fluorescent intensity of phosphorylated Serine 5 on RNA-polymerase-2. This phosphorylation occurs shortly after the initiation of transcription, before the capping of the mRNA (3, 4) (Fig. 6A), and strongly correlates with transcriptional activity (5, 6). The intensity of the immunofluorescence of activated RNA-polymerase-2 within each nucleus was measured (Fig. 6B) from ten randomly selected myofibers of 5 patients from the ICU A + group and 5 controls (Table S9). Microscope slides with single myofibers attached to the coverslip were prepared as mentioned above, using the same blocking and permeabilization steps. The fibers were incubated overnight with primary antibodies (Phospho-RNA polymerase II CTD (Serine 5), fast MHC and Lamin A/C (Table S13, #1, #5, #6). Next, the fibers were incubated overnight at 4C with secondary antibodies (Table S14, #1, #2, #3). The next day, the fibers were incubated with pre-conjugated phalloidin (AF405) to stain actin. The fibers were mounted in Vectashield (Vector Labs) and imaged within the following week. The Z-stacks were loaded into Imaris analysis software, and the fibers and nuclei were segmented using the Lamin A/C and phalloidin staining after a background subtraction pre-processing step using automatic thresholding. Each Z-stack was checked for wrongfully segmented nuclei. Phospho-RNA polymerase II CTD (Serine 5) signal intensity was measured within each nucleus and used as a measure of transcriptional activity.

*Single muscle fibers: PCM-1 positive myonuclei*
Microscope slides with single myofibers attached to the coverslip were prepared as mentioned above, using the same blocking and permeabilization steps. The fibers were incubated overnight at 4C with primary antibody PCM-1 (Table S13, #7). Next, the fibers were incubated overnight at 4C with secondary antibody and AF647-conjugated phalloidin, 1:1000 Invitrogen. Next, slides were incubated with DAPI (2µg/mL) for 10 minutes at room temperature and the samples were mounted in Vectashield and imaged within the following week. Myofibers and myonuclei were segmented using Imaris software using automatic thresholding using phalloidin and PCM-1/DAPI staining after a background subtraction pre-processing step. The amount of PCM-1 positive nuclei in each fiber was determined automatically by the software and checked by a researcher blinded to the samples.

*TUNEL-assay*
Cryosections (8 µm thick, perpendicular to myofiber direction) were cut from the frozen biopsies. Apoptotic nuclei were identified by staining DNA breaks present in nuclei using a Click-iT™ plus TUNEL assay kits for in situ apoptosis detection, C10618, Invitrogen, according to the manufacturer’s instructions. After TUNEL-staining, the sections were incubated overnight at 4°C with primary antibodies staining PCM-1 and Dystrophin (Table S13, #7, #8). Next, the sections were incubated with secondary antibodies (Table S14, #5, #6) and incubated with DAPI, Invitrogen for 10 minutes. Per set of staining, a positive control was stained using DNAse-I to induce DNA breaks (supplemental figure 2). The sections were mounted with mowiol 4-88 (Calbiochem 475904) and imaged within the following week on a Zeiss AxioImager Fluorescent widefield microscope with a 20X objective. The images were analyzed using the FIJI plugin Stardist to segment nuclei. A blinded member of the study team manually counted TUNEL-positive nuclei. Each TUNEL-positive nucleus was then assessed for PCM-1 positive staining and positioning within the dystrophin barrier. Total myonuclear counts were determined using the FIJI plugin Stardist for automatic nuclear segmentation, then scored manually using PCM-1 staining. All automatically segmented images were visually inspected for accuracy and adjusted accordingly.

*Caspase-3 staining*
Cryosections (8 µm thick, perpendicular to myofiber direction) were cut from the frozen biopsies. The sections were incubated overnight at 4°C with primary antibodies staining PCM1, Cleaved caspase-3 and Laminin (Table S13 #9, #10, #11). Next, the sections were incubated with secondary antibodies (Table S14 #1, #3) and incubated with DAPI, Invitrogen for 10 minutes. The sections were mounted with mowiol and imaged within the following week on a Keyence BZ-X Fluorescent widefield microscope with a 20X objective. Sections were analyzed using FIJI with MuscleJ2 plugin (7). Activated caspase-3 positive nuclei were identified manually and assessed for PCM-1 positive staining and positioning within the laminin barrier. Analysis was performed by a researcher blinded to the samples.

*RNA-sequencing*
Diaphragm biopsies were cut while submerged in liquid nitrogen. A small piece was transferred to an Eppendorf tube and RNA was extracted using a kit (miRNAeasy kit, Qiagen) according to the manufacturer’s instructions. Samples were sent to the Max-Planck-Institute for Heart and Lung Research in Bad Nauheim, Germany. RNA and library preparation integrity were confirmed with a LabChip Gx Touch 24 (Perkin Elmer). RNA integrity was assessed using a Agilent fragment analyzer, only samples with an RQN number >4.6 were further processed for sequencing. Libraries were prepared with 1µg of total RNA using SMARTer Stranded Total RNA Sample Prep Kit - HI Mammalian (Clontech). Sequencing was performed on a NextSeq500 (Illumina) with v2 chemistry, resulting in a minimum of 27M reads per library with a 75bp single-end setup. The resulting raw reads were assessed for quality, adapter content, and duplication rates with FastQC (http://www.bioinformatics.babraham.ac.uk/projects/fastqc). Trimmomatic version 0.39 was used to trim reads after a quality drop below a mean of Q20 in a window of 10 nucleotides. Only reads between 30 and 75 nucleotides were used in subsequent analyses. Trimmed and filtered reads were aligned against the human Ensembl genome version hg38 (GRC38.27) using STAR 2.6.1d with the parameter “-- outFilterMismatchNoverLmax 0.1” to increase the maximum ratio of mismatches to mapped length to 10%. The number of reads aligning to genes was counted using the featureCounts 1.6.5 tool from the Subread package. Only reads mapping at least partially inside exons were admitted and aggregated per gene. Reads overlapping multiple genes or aligning to multiple regions were excluded. Differentially expressed © 2020 American Medical Association. All rights reserved. genes were identified using DESeq2 version 1.26. The Ensemble annotation was enriched with UniProt data (release 06.06.2014) based on Ensembl gene identifiers (Activities at the Universal Protein Resource (UniProt)). GO-term analysis was performed with Metascape. The function of genes was referred to the “NCBI’s Gene” and “GeneCards” databases*.*

*PAX7 staining*
Cryosections (8 µm thick, perpendicular to myofiber direction) were cut from the frozen biopsies. Sections were air-dried and fixed with acetone. Next, the sections were incubated with PAX-7, laminin, and horse anti-mouse biotinylated (Table S13, #12, #13, Then, sections were incubated with secondary antibodies (Table S14, #5, #8), and Hoechst (Life Technologies, H3569, 1:15000). The sections were mounted with mowiol and imaged within the following week on a Keyence BZ-X Fluorescent widefield microscope with a 20X objective. The PAX-7 positive cells were counted by hand.

*Statistical analysis*

Normality of the distribution of the studied variables was assessed visually on normal probability plots. Log transformation was performed if necessary. To compare the difference between ICU patients and control patients, student’s t-test or Mann-Whitney-U was applied for non-repeat measurements; and linear mixed model with patients as the random factor was applied for measurements involving technical replicates in all human samples. For linear mixed models, Greenhouse-Geisser correction was applied to adjust for potential lack of sphericity. For the comparison of three groups or more, one-way analysis of variance (ANOVA) or Kruskal-Wallis tests were performed with Tukey’s or Dunn’s post-hoc tests, depending on the distribution of the data. We used a two-sided significance level of 5% for all analyses. Unless otherwise noted, data are expressed as mean (± standard error), median [interquartile range], or frequencies (percentage), as appropriate.

**Supplementary results**

*Relationship between myofiber CSA and myonuclear number*

When we examined the relationship between myofiber CSA and myonuclear number in isolated diaphragm myofibers, we found a comparable positive correlation in all groups (Fig. S12A). Myonuclear domain size and myofiber CSA showed a similar positive correlation, but the elevation of the regression line in the ICU group with atrophy was significantly lower, indicating smaller myonuclear domains for the same CSA (Fig. S12B, left). The slope of the regression line of the isolated diaphragm myofibers of ICU patients without atrophy had a significantly steeper relationship between myonuclear domain and cross-sectional area compared to the ICU group without atrophy (Fig. S12B, right).

**Supplementary references in the main text**

S1 Claassen WJ, van der Berg M, Baelde RR, Bogaards S, Bonis L, Hakkeling H, et al. Myonuclear apoptosis underlies diaphragm atrophy in mechanically ventilated ICU patients. medRxiv. 2024:2024.07.23.24310792.

S2 Claassen WJ, Kirby TJ, Heunks LM, Ottenheijm CA. ICU‑acquired diaphragm weakness: the role of the myonucleus. Intensive Care Med Exp. 2022(10 (suppl 2)):257-8.

S3 Vitale I, Pietrocola F, Guilbaud E, Aaronson SA, Abrams JM, Adam D, et al. Apoptotic cell death in disease-Current understanding of the NCCD 2023. Cell Death Differ. 2023;30(5):1097-154.

S4 Lossi L. The concept of intrinsic versus extrinsic apoptosis. Biochem J. 2022;479(3):357-84.

S5 Gaulton N, Wakelin G, Young LV, Wotherspoon S, Kamal M, Parise G, et al. Twist2-expressing cells reside in human skeletal muscle and are responsive to aging and resistance exercise training. FASEB J. 2022;36(12):e22642.

S6 Flynn CGK, Ginkel PRV, Hubert KA, Guo Q, Hrycaj SM, McDermott AE, et al. Hox11-expressing interstitial cells contribute to adult skeletal muscle at homeostasis. Development. 2023;150(4).

S7 Li K, van Delft MF, Dewson G. Too much death can kill you: inhibiting intrinsic apoptosis to treat disease. EMBO J. 2021;40(14):e107341.

S8 Dhani S, Zhao Y, Zhivotovsky B. A long way to go: caspase inhibitors in clinical use. Cell Death Dis. 2021;12(10):949.

S9 Powers SK, Hudson MB, Nelson WB, Talbert EE, Min K, Szeto HH, et al. Mitochondria-targeted antioxidants protect against mechanical ventilation-induced diaphragm weakness. Crit Care Med. 2011;39(7):1749-59.

S10 Schaaf GJ, Canibano-Fraile R, van Gestel TJM, van der Ploeg AT, Pijnappel W. Restoring the regenerative balance in neuromuscular disorders: satellite cell activation as therapeutic target in Pompe disease. Ann Transl Med. 2019;7(13):280.

S11. Heunks L, Donker DW, Oppersma E, Ottenheijm CAC, Doorduin J. Diaphragm Pacing in Early Critical Illness? A Plea for a Super-Relaxed Approach. Am J Respir Crit Care Med. 2025;211(3):316-8.

S12. Dres M, de Abreu MG, Merdji H, Muller-Redetzky H, Dellweg D, Randerath WJ, et al. Randomized Clinical Study of Temporary Transvenous Phrenic Nerve Stimulation in Difficult-to-Wean Patients. Am J Respir Crit Care Med. 2022;205(10):1169-78.

**References of supplement**

1. van den Berg M, Hooijman PE, Beishuizen A, de Waard MC, Paul MA, Hartemink KJ, et al. Diaphragm Atrophy and Weakness in the Absence of Mitochondrial Dysfunction in the Critically Ill. Am J Respir Crit Care Med. 2017;196(12):1544-58.

2. Dorland YL, Cornelissen AS, Kuijk C, Tol S, Hoogenboezem M, van Buul JD, et al. Nuclear shape, protrusive behaviour and in vivo retention of human bone marrow mesenchymal stromal cells is controlled by Lamin-A/C expression. Sci Rep. 2019;9(1):14401.

3. Komarnitsky P, Cho EJ, Buratowski S. Different phosphorylated forms of RNA polymerase II and associated mRNA processing factors during transcription. Genes Dev. 2000;14(19):2452-60.

4. Cho EJ, Takagi T, Moore CR, Buratowski S. mRNA capping enzyme is recruited to the transcription complex by phosphorylation of the RNA polymerase II carboxy-terminal domain. Genes Dev. 1997;11(24):3319-26.

5. Jeronimo C, Bataille AR, Robert F. The writers, readers, and functions of the RNA polymerase II C-terminal domain code. Chem Rev. 2013;113(11):8491-522.

6. Bataille AR, Jeronimo C, Jacques PE, Laramee L, Fortin ME, Forest A, et al. A universal RNA polymerase II CTD cycle is orchestrated by complex interplays between kinase, phosphatase, and isomerase enzymes along genes. Mol Cell. 2012;45(2):158-70.

7. Danckaert A, Trignol A, Le Loher G, Loubens S, Staels B, Duez H, et al. MuscleJ2: a rebuilding of MuscleJ with new features for high-content analysis of skeletal muscle immunofluorescence slides. Skelet Muscle. 2023;13(1):14.

**Supplemental figure legends**

**Figure S1. Relative expression levels of genes associated with the P53 pathway
A:** top 50 DEG heatmap. Top 50 most significantly differentially expressed DEGs for each contrast (sorted by smallest adjusted *p*-value). 1714 genes were significantly upregulated and 1236 genes were significantly downregulated in the ICU group. Genes with a significance level *p*<0.05 and a fold change of >1.5 were deemed differentially expressed. CTRL *N* = 8; ICU *N* = 17. **B:** Relative expression levels of genes associated with the P53 pathway determined with RNA-sequencing. *p* -values were adjusted for repeated testing. CTRL *N* = 8; ICU *N* = 17. **C:** Relative expression levels of PAX7. CTRL *N* = 8; ICU *N* = 17. The ICU groups with and without atrophy were pooled to calculate the significance level. *p* -values were adjusted for repeated testing. CTRL = Control group, ICU = Intensive care patients

**Figure S2. Representative images of DNAse-treated muscle cross-section after TUNEL staining**Note that every nucleus has a positive TUNEL signal after inducing DNA breaks with DNAse treatment. Scale bar is 50 µm.

**Figure S3. Apoptotic index of non-myonuclei
A:** Quantification of TUNEL index for non-myonuclei. The grey bar represents the median value within the groups of patients, brackets represent interquartile ranges. Each colored symbol represents the value of a single patient. ICU A+ *N* = 14; ICU A− *N* = 7; CTRL; *N* = 11. Significance level was calculated using Kruskal-Wallis test. **B:** Quantification of activated caspase-3 index for non-myonuclei. The grey bar represents the median value within the groups of patients. Each colored symbol represents the value of a single patient. ICU A+ *N* = 7; ICU A− *N* = 6; CTRL; *N* = 7   Significance level calculated T-test or Mann-Whitney U test, depending on distribution of the data.

**Figure S4. Increased Caspase-3 index in Quadriceps biopsies of mechanically ventilated patients.**

**A:** Representative images of vastus lateralis muscle cross-sections stained with Cleaved caspase-3 antibody, PCM1 antibody, Laminin antibody and DAPI. Nuclei with a cleaved-Caspase-3 positive signal were designated as apoptotic myonuclei when they were PCM1 positive and were located within the laminin barrier. Top scale bar is 50µm, bottom scale bar is 20 µm. **B:** Quantification of activated caspase-3 index in quadriceps biopsies**,** calculated as the percentage of activated caspase-3-positive myonuclei. Total myonuclear count was determined by counting PCM1-positive nuclei. The grey bar represents the mean value within the groups of patients, brackets represent the standard deviation. Each colored symbol represents the value of a single patient. ICU-Q *N* = 10; CTRL-Q *N* = 5. The significance level was calculated using an unpaired T-test. **C:** Quantification of activated caspase-3 index for non-myonuclei in quadriceps biopsies. The grey bar represents the mean value within the groups of patients, brackets represent the standard deviation. Each colored symbol represents the value of a single patient **D:** Quantification of activated caspase-3 index in the ICU quadriceps and ICU diaphragm biopsies**,** calculated as the percentage of activated caspase-3-positive myonuclei. Total myonuclear count was determined by counting PCM1-positive nuclei. The grey bar represents the median value within the groups of patients. Each colored symbol represents the value of a single patient. ICU Quad *N = 10*; ICU DIA *N* = 13. Significance level was calculated using Kruskal-Wallis test. **E:** Quantification of activated caspase-3 index in all control patients**,** calculated as the percentage of activated caspase-3-positive myonuclei. Total myonuclear count was determined by counting PCM1-positive nuclei. The grey bar represents the median value within the groups of patients. Each colored symbol represents the value of a single patient. . **F:** Mean myofiber CSA in quadriceps biopsies. The grey bars represent the mean value of the groups. Colored symbols represent the value per patient. ICU-Q *N* = 10; CTRL-Q *N* = 5. The significance level was calculated using unpaired T-test. CTRL Quad *N* = 5; CTRL Dia *N* = 7. Significance level calculated with unpaired t-test. ICU; N = 10, CTRL; N = 5. ICU-Q = ICU Quadriceps group, CTRL-Q = Control Quadriceps group, * = *p*<0.05, ** = *p*<0.01

**Figure S4. Increased Caspase-3 index in Quadriceps biopsies of MV patients.**

**A:** Representative images of vastus lateralis muscle cross-sections stained with Cleaved caspase-3 antibody, PCM1 antibody, Laminin antibody and DAPI. Nuclei with a cleaved-Caspase-3 positive signal were designated as apoptotic myonuclei when they were PCM1 positive and were located within the laminin barrier. Top scale bar is 50µm, bottom scale bar is 20 µm. **B:** Quantification of activated caspase-3 index**,** calculated as the percentage of activated caspase-3-positive myonuclei. Total myonuclear count was determined by counting PCM1-positive nuclei. The grey bar represents the mean value within the groups of patients, brackets represent the standard deviation. Each colored symbol represents the value of a single patient. ICU-Q *N* = 10; CTRL-Q *N* = 5. The significance level was calculated using an unpaired T-test. **C:** Quantification of activated caspase-3 index for non-myonuclei. The grey bar represents the mean value within the groups of patients, brackets represent the standard deviation. Each colored symbol represents the value of a single patient. **D:** Mean myofiber CSA The grey bars represent the mean value of the groups. Colored symbols represent the value per patient. ICU-Q *N* = 10; CTRL-Q *N* = 5. The significance level was calculated using unpaired T-test. ICU; N = 10, CTRL; N = 5. ICU-Q = ICU Quadriceps group, CTRL-Q = Control Quadriceps group, * = *p*<0.05, ** = *p*<0.01

**Figure S5. Proportion of PCM-1 + nuclei within manually isolated single myofibers**

**A:** Representative images of a single myofiber stained with DAPI (blue) and PCM1 (yellow). Scale bar is 20 µm. Arrow indicates PCM1-negative nucleus. Note the PCM1 negative nucleus indicated with the white arrow. **B:** Proportion of PCM1 + nuclei within manually isolated myofibers from controls *N* = 3. **C:** Proportion of PCM1 + nuclei within manually isolated myofibers from ICU patients *N* = 3.

**Figure S6. Myofiber volume, myonuclear number and myonuclear domain of isolated myofibers in two ICU groups
A:** Quantification of myofiber volume, calculated as volume per mm fiber was normalized to a sarcomere length of 2.5 µm. Every grey dot represents the value of a single muscle fiber, and the colored symbols represent the mean values of a single patient. Slow-twitch fibers: ICU *N =* 24, *n* = 134; CTRL *N =* 10, *n =* 48. Fast-twitch fibers: ICU *N =* 24, *n =* 104; CTRL *N =* 10, *n =* 46. **C:** Quantification of myonuclear number. Every grey dot represents the value of a single muscle fiber and the colored symbols represent the mean values of a single patient. Slow-twitch fibers: ICU *N =* 24, *n* = 134; CTRL *N =* 10, *n =* 48. Fast-twitch fibers: ICU *N =* 24, *n =* 104; CTRL *N =* 10, *n =* 46. **D:** Quantification of myonuclear domain size. Every grey dot represents the value of a single muscle fiber and the colored symbols represent the mean values of a single patient. Significance levels were calculated using linear mixed models with the patients as the random factor. Black bars indicate the median of the whole group. Slow-twitch fibers: ICU *N =* 24, *n* = 134; CTRL *N =* 10, *n =* 48. Fast-twitch fibers: ICU *N =* 24, *n =* 104; CTRL *N =* 10, *n =* 46. ICU = ICU group; CTRL = Control group. * denotes p < 0.05; ** denotes p < 0.01. N = number of patients, n = number of analyzed myofibers.

**Figure S7. Myonuclear content of diaphragm and quadriceps cross-sections
A:**  Representative images of diaphragm cross-sections with DAPI (blue) PCM1 (yellow) and Dystrophin (grey). Scale bar is 50 µm. **B:** Myonuclear number per myofiber. The grey bars represent the mean value of the groups. Colored symbols represent the value per patient. CTRL = 8; ICA A- = 7; ICA A+ = 11. Statistics are performed using one-way ANOVA. Post-hoc testing with Tukey’s test. CTRL = Control group, ICU A+ = Intensive care patients with myofiber atrophy, ICU A− = Intensive care patients without atrophy, **C:** Myonuclear number per myofiber. The grey bars represent the mean value of the groups. Colored symbols represent the value per patient. CTRL QUAD = control group quadriceps biopsies, ICU QUAD = ICU group, quadriceps biopsies. Statistics are performed using a T-test. ** = *p*<0.01, ****= *p*<0.0001

**Figure S6. Myofiber volume, myonuclear number and myonuclear domain of isolated diaphragm myofibers in ICU patients and controls
A:** Quantification of myofiber volume, calculated as volume per mm fiber was normalized to a sarcomere length of 2.5 µm. Every grey dot represents the value of a single muscle fiber, and the colored symbols represent the mean values of a single patient. Slow-twitch fibers: ICU *N =* 24, *n* = 134; CTRL *N =* 10, *n =* 48. Fast-twitch fibers: ICU *N =* 24, *n =* 104; CTRL *N =* 10, *n =* 46. **B:** Quantification of myonuclear number. Every grey dot represents the value of a single muscle fiber and the colored symbols represent the mean values of a single patient. Slow-twitch fibers: ICU *N =* 24, *n* = 134; CTRL *N =* 10, *n =* 48. Fast-twitch fibers: ICU *N =* 24, *n =* 104; CTRL *N =* 10, *n =* 46. **C:** Quantification of myonuclear domain size. Every grey dot represents the value of a single muscle fiber and the colored symbols represent the mean values of a single patient. Significance levels were calculated using linear mixed models with the patients as the random factor. Black bars indicate the median of the whole group. Slow-twitch fibers: ICU *N =* 24, *n* = 134; CTRL *N =* 10, *n =* 48. Fast-twitch fibers: ICU *N =* 24, *n =* 104; CTRL *N =* 10, *n =* 46. ICU = ICU group; CTRL = Control group. * denotes p < 0.05; ** denotes p < 0.01. N = number of patients, n = number of analyzed myofibers.

**Figure S7. Myonuclear content of diaphragm and quadriceps cross-sections
A:**  Representative images of diaphragm cross-sections with DAPI (blue) PCM1 (yellow) and Dystrophin (grey). Scale bar is 50 µm. **B:** Myonuclear number per myofiber. The grey bars represent the mean value of the groups. Colored symbols represent the value per patient. CTRL = 8; ICA A- = 7; ICA A+ = 11. Statistics are performed using one-way ANOVA. Post-hoc testing with Tukey’s test. CTRL = Control group, ICU A+ = Intensive care patients with myofiber atrophy, ICU A− = Intensive care patients without atrophy, **C:** Myonuclear number per myofiber. The grey bars represent the mean value of the groups. Colored symbols represent the value per patient. CTRL QUAD = control group quadriceps biopsies, ICU QUAD = ICU group, quadriceps biopsies. Statistics are performed using a T-test. ** = *p*<0.01, ****= *p*<0.0001

**Figure S8. Correlations of duration of mechanical ventilation and duration of diaphragm inactivity with myonuclear number and TUNEL, caspase-3 Indices.**

**A:** Correlation of myonuclear number with duration of mechanical ventilation (top) and diaphragm inactivity calculated as time on controlled mechanical ventilation minus time with diaphragm activity (bottom). Correlation coefficient calculated using Spearman r. **B:** Correlation of TUNEL index with duration of mechanical ventilation (top) and diaphragm inactivity calculated as time on controlled mechanical ventilation minus time with diaphragm activity (bottom). Correlation coefficient calculated using Pearson r. **C:** Correlation of TUNEL index with duration of mechanical ventilation (top) and diaphragm inactivity calculated as time on controlled mechanical ventilation minus time with diaphragm activity (bottom). Correlation coefficient calculated using Spearman r.

**Figure S9. Correlations of respiratory outcomes with myonuclear number of myofibers.**

**A:** Correlation of myonuclear number with time to first spontaneous breathing trial, *N* = 18. Correlation coefficients calculated using Spearman r. **B:** Correlation of myonuclear number with time to liberation from the ventilator, *N* = 15. . Correlation coefficient calculated using Spearman r. **C:** Correlation of myonuclear number with duration of mechanical ventilation, all patients, *N = 22*. **D:** Correlation of myonuclear number with duration of mechanical ventilation, patients with <100 hours of mechanical ventilation, *N* = 13. Correlation coefficient calculated using Spearman r.

**Figure S10. Relative expression levels of genes associated with the P53 pathway in ICU patients with and without atrophy.
A:** Relative expression levels of genes associated with the P53 pathway determined with RNA-sequencing. *p* -values were adjusted for repeated testing. CTRL *N* = 8; ICU A+ *N* = 9, ICU A- *N* = 8. **B:** Relative expression levels of PAX7. CTRL *N* = 8; ICU A+ *N* = 9, ICU A- *N* = 8. *p* -values were adjusted for repeated testing. CTRL = Control group, ICU A+ = Intensive care patients with atrophy, ICU A- = ICU patients without atrophy.

**Figure S11. Correlations of plasma C-reactive protein and leucocyte counts with myonuclear number of myofibers and apoptotic indices.**

**A:** Correlation of myonuclear number (*N* = 22, top), TUNEL index *(N=18,* middle) and activated caspase-3 index (*N=13,* bottom) with plasma levels of C-reactive protein (CRP). Correlation coefficients calculated using Pearson r. **B:** Correlation of myonuclear number (*N* = 22, top), TUNEL index *(N=18,* (middle) and activated caspase-3 index (*N=13,* bottom) with leucocyte count. Correlation coefficients calculated using Pearson r.

**Figure S12. Scatterplots of nuclear number vs. myofiber cross-sectional area and myonuclear domain vs. cross-sectional area.
A:** Number of nuclei plotted against fiber CSA of every fiber of the ICU A+ (dark red) and ICU A− (light red) groups, compared with the CTRL (blue) group. Slow-twitch fibers: ICU A+ *N =* 14, *n* = 84; ICU A− *N =* 10, *n =* 50; CTRL *N =* 10, *n =* 48. Fast-twitch fibers: ICU A+ *N =* 14, *n =* 56; ICU A− *N =* 10, *n =* 48; CTRL *N =* 10, *n =* 46. R-squared and *p*-value were calculated using simple linear regression. The slope and elevation were compared between the groups using Analysis of Covariance (ANCOVA). **B:** Myonuclear domain plotted against fiber CSA of every fiber of the ICU A+ (dark red) and ICU A− (light red) groups, compared with the CTRL (blue) group. R-squared and *p*-value were calculated using simple linear regression. The slope and elevation were compared between the groups using Analysis of Covariance (ANCOVA). Slow-twitch fibers: ICU A+ *N =* 14, *n* = 84; ICU A− *N =* 10, *n =* 50; CTRL *N =* 10, *n =* 48. Fast-twitch fibers: ICU A+ *N =* 14, *n =* 56; ICU A− *N =* 10, *n =* 48; CTRL *N =* 10, *n =* 46. ICU A+ = ICU group with atrophy, ICU A− = ICU group without atrophy, CTRL = Control group, MND = Myonuclear domain, * = *p*<0.05, ** = *p*<0.0

**Supplementary tables.**

**Supplementary table 1. Experiments performed per diaphragm biopsy.**

| Subject # | Group | Nuclear number and MND | Transcr. activity | RNA-seq | Apoptosis  TUNEL | Apoptosis  Casp3 | PAX 7 |
| --- | --- | --- | --- | --- | --- | --- | --- |
| 25 | ICU A+ | 1 | 0 | 1 | 0 | 0 | 0 |
| 46 | ICU A+ | 1 | 0 | 0 | 0 | 0 | 0 |
| 42 | ICU A+ | 0 | 0 | 1 | 0 | 0 | 0 |
| 50 | ICU A+ | 0 | 0 | 0 | 1 | 0 | 0 |
| 52 | ICU A+ | 1 | 0 | 1 | 0 | 0 | 0 |
| 64 | ICU A+ | 0 | 0 | 1 | 1 | 0 | 1 |
| 72 | ICU A+ | 1 | 0 | 0 | 1 | 1 | 1 |
| 73 | ICU A+ | 1 | 1 | 0 | 1 | 0 | 1 |
| 74 | ICU A+ | 1 | 1 | 1 | 1 | 1 | 1 |
| 78 | ICU A+ | 1 | 0 | 0 | 1 | 1 | 0 |
| 80 | ICU A+ | 1 | 0 | 0 | 1 | 1 | 0 |
| 82 | ICU A+ | 1 | 0 | 1 | 1 | 1 | 0 |
| 89 | ICU A+ | 1 | 1 | 0 | 0 | 0 | 1 |
| 91 | ICU A+ | 1 | 0 | 0 | 1 | 1 | 0 |
| 94 | ICU A+ | 1 | 0 | 1 | 1 | 0 | 0 |
| 99 | ICU A+ | 1 | 1 | 1 | 1 | 1 | 1 |
| 100 | ICU A+ | 1 | 1 | 0 | 1 | 1 | 1 |
| 101 | ICU A+ | 0 | 0 | 1 | 0 | 0 | 0 |
| 3 | ICU A - | 0 | 0 | 0 | 1 | 0 | 0 |
| 12 | ICU A - | 1 | 0 | 0 | 1 | 1 | 0 |
| 15 | ICU A - | 1 | 0 | 0 | 0 | 0 | 0 |
| 22 | ICU A - | 1 | 0 | 0 | 1 | 1 | 0 |
| 27 | ICU A - | 1 | 0 | 1 | 0 | 0 | 0 |
| 38 | ICU A - | 0 | 0 | 1 | 0 | 0 | 0 |
| 57 | ICU A - | 0 | 0 | 1 | 0 | 0 | 0 |
| 66 | ICU A - | 1 | 0 | 0 | 1 | 1 | 0 |
| 70 | ICU A - | 1 | 0 | 1 | 1 | 1 | 0 |
| 77 | ICU A - | 1 | 0 | 1 | 1 | 1 | 0 |
| 83 | ICU A - | 1 | 0 | 0 | 1 | 0 | 0 |
| 95 | ICU A - | 0 | 0 | 1 | 0 | 0 | 0 |
| 96 | ICU A - | 1 | 0 | 0 | 1 | 1 | 0 |
| 97 | ICU A - | 0 | 0 | 1 | 0 | 0 | 0 |
| 6 | CTRL | 0 | 0 | 1 | 0 | 0 | 0 |
| 7 | CTRL | 0 | 0 | 1 | 0 | 0 | 0 |
| 8 | CTRL | 1 | 0 | 0 | 1 | 1 | 0 |
| 41 | CTRL | 1 | 1 | 1 | 0 | 1 | 1 |
| 43 | CTRL | 1 | 0 | 1 | 1 | 1 | 1 |
| 45 | CTRL | 0 | 0 | 1 | 0 | 0 | 0 |
| 48 | CTRL | 1 | 1 | 0 | 1 | 1 | 1 |
| 49 | CTRL | 1 | 1 | 0 | 1 | 1 | 1 |
| 55 | CTRL | 1 | 0 | 0 | 1 | 1 | 1 |
| 56 | CTRL | 1 | 0 | 0 | 1 | 0 | 1 |
| 58 | CTRL | 1 | 1 | 1 | 1 | 1 | 1 |
| 60 | CTRL | 0 | 0 | 1 | 0 | 0 | 0 |
| 62 | CTRL | 1 | 1 | 1 | 1 | 0 | 0 |
| 63 | CTRL | 1 | 0 | 0 | 0 | 0 | 1 |
| 79 | CTRL | 0 | 0 | 1 | 0 | 0 | 0 |

ICU A+ = ICU group with atrophy, ICU A− = ICU group without atrophy, CTRL = Control group.

**Supplementary table 2. Patient characteristics, RNA-sequencing experiment**

|  | Control (n=9) | ICU (n=17) | P-value |
| --- | --- | --- | --- |
| Age (years) | 64 [51-71] | 66 [42-75] | 0.968 |
| M (%) | 7 (78) | 8 (47) | 0.132 |
| BMI (Kg/m^2^) | 24 [23-30] | 25 [23-28] | 0.804 |
| APACHE-3 | - |  | - |
| Ventilation (hours) | 1.3 [0.9-2] | 58 [42-169] | <0.001 |
| Myofiber CSA (μm^2^) | 3021 [2476-3542] | 2067 [1729-3039] | 0.099 |

*BMI = Body Mass Index APACHE = Acute Physiology And Chronic Health Evaluation, CSA = Cross-Sectional Area. Data shown as Median [IQR]. P-values of continuous data calculated with unpaired T-test or Mann-Whitney-U test, depending on distribution of the data. P-values of categorical data calculated with Chi-squared test.*

**Supplementary table 3. Patient characteristics, TUNEL experiment**

|  | Control (n=8) | ICU (n=18) | P-value |
| --- | --- | --- | --- |
| Age (years) | 67 [58-73] | 66 [50-72] | 0.676 |
| M (%) | 3 (38) | 5 (28) | 0.620 |
| BMI (Kg/m^2^) | 27 [24-29] | 27 [23-30] | 0.380 |
| APACHE-3 | - | 66 [26-104] | - |
| Ventilation (hours) | 1.5 [0.9-2.0] | 99 [56-205] | <0.001 |
| Myofiber CSA(μm^2^) | 2147 [1624-2816] | 1563 [1064-3366] | 0.812 |

*BMI = Body Mass Index APACHE = Acute Physiology And Chronic Health Evaluation, CSA = Cross-Sectional Area. Data shown as Median [IQR]. P-values of continuous data calculated with unpaired T-test or Mann-Whitney-U test, depending on distribution of the data. P-values of categorical data calculated with Chi-squared test.*

**Supplementary table 4. Patient characteristics, Caspase-3 staining experiment.**

|  | Control (n=7) | ICU (n=14) | P-value |
| --- | --- | --- | --- |
| Age (years) | 66 [60-71] | 62 [47-70] | 0.456 |
| M (%) | 3 (57) | 5 (38) | 0.751 |
| BMI (Kg/m^2^) | 26 [24-28] | 25 [22-28] | 0.864 |
| APACHE-3 | - | 66 [37-96] | - |
| Ventilation (hours) | 1.0 [0.9-1.8] | 65 [36-205] | <0.001 |
| Myofiber CSA (μm^2^) | 2501 [1720-3024] | 1684 [1064-3464] | 0.502 |

*BMI = Body Mass Index APACHE = Acute Physiology And Chronic Health Evaluation, CSA = Cross-Sectional Area. Data shown as Median [IQR]. P-values of continuous data calculated with unpaired T-test or Mann-Whitney-U test, depending on distribution of the data. P-values of categorical data calculated with Chi-squared test.*

**Supplementary table 5. Patient characteristics, quadriceps experiment**

|  | Control (n=5) | ICU quadriceps (n=10) | P-value |
| --- | --- | --- | --- |
| Age (years) | 51 [21-58] | 63 [32-82] | 0.148 |
| M (%) | 4 (80) | 9 (90) | 0.591 |
| BMI (Kg/m^2^) | 25 [23-28] | 23 [22-29] | 0.864 |
| APACHE-4 | - | 97 [73-130] | - |
| Ventilation (hours) | - | 39 [17-50] | - |
| Myofiber CSA (μm^2^) | 6509 [4828-9339] | 4438 [3723-5816] | 0.199 |

*BMI = Body Mass Index APACHE = Acute Physiology And Chronic Health Evaluation, CSA = Cross-Sectional Area. Data shown as Mean ± SD or Median [IQR], depending on distribution of the data. P-values of continuous data calculated with unpaired T-test or Mann-Whitney-U test, depending on distribution of the data. P-values of categorical data calculated with Chi-squared test*

**Supplementary table 6. Patient characteristics, diaphragm cohort compared to quadriceps cohort.**

| Characteristic | DIA ICU Patients (n=24) | | QUAD ICU  (n=10) | | P-value |
| --- | --- | --- | --- | --- | --- |
| Age (years) | 66 [50-73] | | 63 [32-82] | | 0.985 |
| Male (%) | 15 (63) | | 9 (90) | | 0.109 |
| BMI (Kg/m^2^) | 25 [21-28] | | 23 [22-29] | | 0.940 |
| APACHE-3 | 73 [47-111] | | 97 [73-130] | | 0.147 |
| Ventilation (hours) | 66 [44-193] | | 39 [17-50] | | 0.010 |
| Myofiber CSA (μm^2^) | 1736 [1250-3035] | | 4438 [3723-5816] | | <0.001 |
| Medical history, n (%) | |  | |  | |
| Smoking | 13 (54) | | 4 (40) | | 0.452 |
| COPD ≤ G2 | 3 (13) | | 1 (10) | | 0.837 |
| Other lung disease | 1 (4) | | 0 (0) | |  |
| Cardiac | 3 (13) | | 0 (0) | |  |
| Arterial vascular disease | 14 (58) | | 1 (10) | | 0.010 |
| Hypertension | 9 (38) | | 1 (10) | |  |
| CKD | 2 (8) | | 0 (0) | |  |
| T2DM | 2 (8) | | 0 (0) | |  |
| Hypothyroidism | 2 (8) | | 0 (0) | |  |
| Malignancy lung | 1 (4) | | 0 (0) | |  |
| Malignancy other | 2 (1) | | 0 (0) | |  |
| Medication | |  | |  | |
| Steroids | 18 (75) | | 4 (40) | | 0.052 |
| Neuromuscular blockers | 12 (50) | | 3 (30) | | 0.285 |
| Vasopressors | 19 (79) | | 8 (80) | | 0.956 |

Data displayed as Median [IQR], p-values calculated with Mann-Whitney-U or chi-squared tests BMI: Body Mass Index, MVhr: duration of mechanical ventilation before biopsy *BMI = Body Mass Index APACHE = Acute Physiology And Chronic Health Evaluation, CSA = Cross-Sectional Area, COPD = Chronic Obstructive Pulmonary Disease, CKD = Chronic Kidney Disease, T2DM = Type 2 Diabetes Mellitus, CID = Chronic Inflammatory Disease, P/F-ratio = arterial partial pressure of oxygen (PaO2) divided by the inspired oxygen concentration (FiO2), A-a gradient = alveolar-arterial gradient. Data shown as Median [IQR]. P-values of continuous data calculated with one-way analysis of variance or Kruskal-Wallis test, depending on distribution of the data. P-values of categorical data calculated with Chi-squared test. * indicates a significant difference with the control group calculated with post-hoc tests.*

**Supplementary table 7. PCM-1 staining of manually isolated myofibers.**

|  | Control (n=3) | ICU (n=3) | P-value |
| --- | --- | --- | --- |
| Age | 64 [61-68] | 71 [61-75] | 0.376 |
| Male (%) | 2 (60) | 2 (60) | 1.0 |
| BMI | 31 [29-33] | 25 [20-28] | 0.100 |
| APACHE | - | 87 [57-149] | - |
| Ventilation (hours) | 1.0 [0.8-2.0] | 45 [24-84] | 0.400 |
| Myofiber CSA (μm^2^) | 3128 [2437-3132] | 1065 [530-1628] | 0.100 |

*BMI = Body Mass Index APACHE = Acute Physiology And Chronic Health Evaluation, CSA = Cross-Sectional Area*

**Supplementary table 8. Additional clinical characteristics of ICU patients with and without atrophy**

|  | ICU A+ (n=17) | ICU A- (n=10) | P-value |
| --- | --- | --- | --- |
| Arterial blood gas analysis, median [IQR] |  |  |  |
| pH (min) | 7.14 [7.05-7.32] | 7.27 [7.18-7.33] | 0.368 |
| pO_2_ (min), mmHg | 60.8 [42.0-74.0] | 58.5 [44.8-70.3] | 0.966 |
| pCO_2_ (max), mmHg | 45.0 [34.2-55.1] | 56.9 [44.8-70.3] | 0.087 |
| Bicarbonate (min), mmol/l | 14.0 [10.0-17.0] | 18.0 [13.0-21.1] | 0.127 |
| Lactate (max), mmol/l | 7.7 [3.2-10.1] | 3.7 [1.4-16.8] | 0.521 |
| P/F-ratio (min) | 135 [98.0-287] | 146 [91.0-246] | 0.792 |
| A-a gradient (max) | 197 [101-251] | 247 [144-375] | 0.335 |
| Leucocyte count at time of biopsy (x10^9/L) | 13.4 [9.3-21.3] | 13.8 [6.5-19.9] | 0.512 |
| CRP at time of biopsy (mg/mL) | 197.1 [162-342] | 199.5 [144-287] | 0.912 |
| Septic shock*, n (%) | 9 (53) | 4 (40) | 0.516 |
| Days in septic shock*, median [IQR] | 2 [0-3] | 1 [0-5] | 0.671 |
| Died on ICU (%) | 6 (35) | 3 (30) | 0.778 |

*Data are represented median [IQR]. P/F-ratio = arterial partial pressure of oxygen (PaO2) divided by the inspired oxygen concentration (FiO2), A-a gradient = alveolar-arterial gradient. *Data on septic shock were retrieved retrospectively. (Days in) Septic shock before biopsy data is defined as the presence ≤2 SIRS criteria, an infection focus and the need for vasopressors or a mean arterial blood pressure <65 or a systolic blood pressure <90.*

**Supplementary table 9. Patient characteristics, transcriptional activity of nuclei experiment**

|  | Control (n=5) | ICU (n=5) | P-value |
| --- | --- | --- | --- |
| Age | 64 [61-68] | 62 [61-71] | 1.0 |
| Male (%) | 3 (60) | 3 (60) | 1.0 |
| BMI | 29 [27-31] | 25 [20-25] | 0.032 |
| APACHE-3 | - | 57 [53-87] | - |
| Ventilation (hours) | 1.0 [0.6-1.5] | 63 [45-84] | 0.008 |
| Myofiber CSA (μm^2^) | 2501 [1975-3129] | 1318 [797-17] | 0.032 |

*BMI = Body Mass Index APACHE = Acute Physiology And Chronic Health Evaluation, CSA = Cross-Sectional Area. Data shown as Median [IQR]. P-values of continuous data calculated with unpaired T-test or Mann-Whitney-U test, depending on distribution of the data. P-values of categorical data calculated with Chi-squared test.*

**Supplementary table 10. Patient characteristics, PAX-7 staining experiment.**

|  | Control (n=8) | ICU (n=8) | P-value |
| --- | --- | --- | --- |
| Age (years) | 64 [58-73] | 69 [60-73] | 0.798 |
| M (%) | 5 (63) | 5 (63) | 1.0 |
| BMI (Kg/m^2^) | 28 [25-30] | 25 [22-29] | 0.328 |
| APACHE-3 | - | 72 [15-124] | - |
| Ventilation (hours) | 1.3 [0.8-1.5] | 74 [49-131] | <0.001 |
| Myofiber CSA (μm^2^) | 2180 [1667-2856] | 1192 [952-1709] | 0.015 |
|  |  |  |  |

*BMI = Body Mass Index APACHE = Acute Physiology And Chronic Health Evaluation, CSA = Cross-Sectional Area. Data shown as Median [IQR]. P-values of continuous data calculated with unpaired T-test or Mann-Whitney-U test, depending on distribution of the data. P-value of categorical data calculated with Chi-squared test.*

**Supplementary table 12. Composition of solutions**

| Solutions | Composition |
| --- | --- |
| Relaxing solution | 100 mM BES, 14.5 mM creatine phosphate, 6.97 mM EGTA, 40.76 mM K-propionate, 6.48 mM MgCl_2_, 5.89 mM Na_2_-ATP, and low concentration of freshly added protease inhibitors. |

**Supplementary table 13. Primary antibodies**

| # | Antigen | Name | Type | Dilution | Source | Code |
| --- | --- | --- | --- | --- | --- | --- |
| 1 | MYH 1 | MY-32 | Mouse mono IgG1 | 1:50 | Abcam | ab51263 |
| 2 | Lamin A/C | E1 | Mouse mono IgG1 | 1:200 | Santa Cruz | SC-376248 |
| 3 | MYH pan | A4.1025 | Mouse mono IgG2a | 1:600 | DSHB | A4.1025 |
| 4 | MYH 1 | MY-32 | Rabbit poly IgG | 1:1000 | Abcam | ab91506 |
| 5 | RNA-pol-II (Ser5) | 4H8 | Mouse mono IgG1 | 1:100 | Thermo | MA1-46093 |
| 6 | Lamin A/C | 4C11 | Mouse mono IgG2a | 1:200 | Cell signaling | 4777S |
| 7 | PCM1 | HPA023370 | Rabbit poly IgG | 1:2500 | Sigma | HPA023370 |
| 8 | Dystrophin | MANDRA1 7A10 | Mouse mono IgG1 | 1:400 | DSHB | MANDRA1 7A10 |
| 9 | PCM1 | G6 | Mouse mono IgG1 | 1:200 | Santa Cruz | sc398365 |
| 10 | Cleaved Casp-3 | Asp-175 | Rabbit poly IgG | 1:400 | Cell signaling | 9661 |
| 11 | Laminin | D18 | Mouse mono IgG2a | 1:200 | DSHB | D18 |
| 12 | PAX7 | PAX7 | Mouse mono IgG1 | 1:15 | DSHB | PAX7 |
| 13 | Laminin | L9393 | Rabbit poly IgG | 1:500 | Sigma | L9393) |

**Supplementary table 14. Secondary antibodies**

| # | Reactivity | Animal | Type | Dilution | Source | Code |
| --- | --- | --- | --- | --- | --- | --- |
| 1 | Mouse IgG1 | Goat | AF 555 | 1:100 | Invitrogen | A21127 |
| 2 | Mouse IG2a | Goat | AF 488 | 1:100 | Invitrogen | A21131 |
| 3 | Rabbit IgG (H+L) | Donkey | AF647 | 1:100 | Invitrogen | A21447 |
| 4 | Rabbit IgG (H+L) | Donkey | AF555 | 1:100 | Invitrogen | A21432 |
| 5 | Rabbit IgG (H+L) | Donkey | AF488 | 1:100 | Invitrogen | A11055 |
| 6 | Mouse IgG (H+L) | Donkey | AF647 | 1:100 | Invitrogen | A31574 |
| 7 | Mouse IgG | Horse | Biotinylated | 1:50 | Vectorlabs | BA2000 |
| 8 | Streptavidin | - | AF594 | 1:500 | Thermo | S32356 |
